# Supplementary material for: Glis2 is an early effector of polycystin signaling and a target for therapy in polycystic kidney disease
Source: Nat Commun. 2024 May 1;15:3698. doi: 10.1038/s41467-024-48025-6 (PMC11063051; doi:10.1038/s41467-024-48025-6)
Supplement: Supplementary file 1 — Supplementary Information [file 41467_2024_48025_MOESM1_ESM.pdf]

Supplementary Information

***Glis2* is an early effector of polycystin signaling and a target for therapy in polycystic kidney disease**

Chao Zhang[1*], Michael Rehman[1*], Xin Tian[1*], Steven Lim Cho Pei[1], Jianlei Gu[2], Thomas A. Bell 3rd[3], Ke Dong[1], Ming Shen Tham[1], Yiqiang Cai[1], Zemeng Wei[1], Felix Behrens[1], Anton M. Jetten[4], Hongyu Zhao[2,5,6], Monkol Lek[2], Stefan Somlo[1,2,‡]

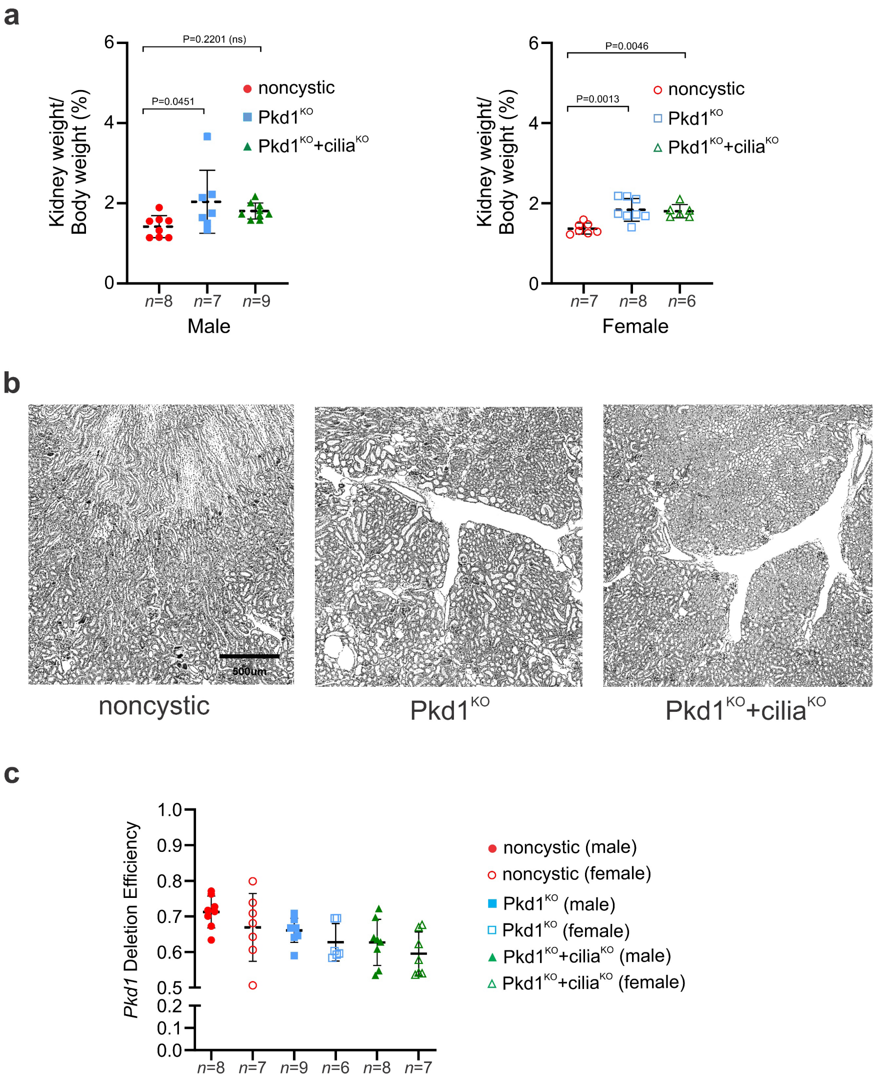

Supplementary Figure 1: Early stage Pkd1^{KO} and Pkd1^{KO}+cilia^{KO} show mild tubule dilation without cyst formation. a, Quantitative data showing the kidney weight-to-body weight ratio from 7 weeks-old mice of both sexes. Noncystic (red circle), Pkd1^{KO} (blue square) and Pkd1^{KO}+cilia^{KO} (green triangle). All mice received doxycycline from P28–42. Male mice filled symbols; female mice, open symbols. '*n*', number of mice in each group. Multiple-group comparisons were performed using one-way ANOVA followed by Tukey's multiple-comparison test and are presented as the mean ± s.e.m. **b,** Representative images of kidneys from 7 weeks-old male mice with the indicated genotypes. Scale bar = 500 µm. At least one section from 3 kidneys for each group were examined for all representative images. **c,** The aggregate semi-quantitative data showing the *Pkd1*^{fl} allele deletion efficiency (see Methods) in kidney from mice with the indicated genotypes and sex. These are the same mice used for the TRAP studies and colors and *n*, symbol shapes correspond to genotype and sex denoted in **a**. Source data for exact values are provided as a Source Data file.

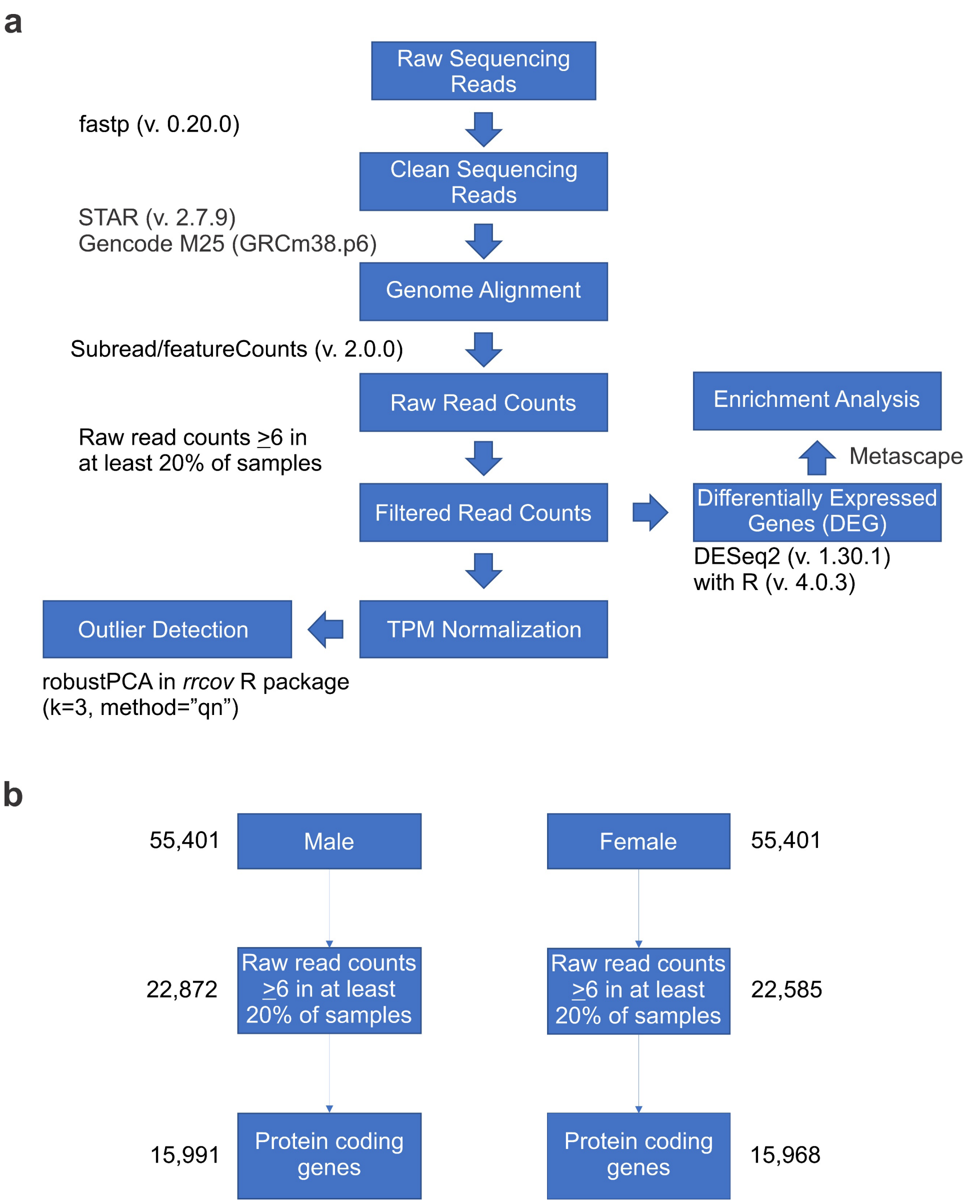

Supplementary Figure 2: Flow diagram for TRAP RNASeq analysis. a, Raw sequencing reads were trimmed using Fastp tool. The trimmed sequencing reads were aligned to the mouse reference genome using STAR. Gene-level expression quantification was performed using Subread/featureCount. Non-expressed/low-expressed genes were filtered using the criteria that the raw read counts of each gene should be no less than 6 in at least 20% of samples. The filtered read counts matrix was normalized using the TPM method (Transcripts Per Million). The filtered read counts matrix was also used to identify DEGs with R package DESeq2. The TPM normalized relative expression matrix was used to perform classic Principal Component Analysis (PCA) and robust PCA analysis to identify potential problematic samples (Supplementary Figure 3). **b**, The steps used to filter out non/low-expressed genes and non-coding genes in male and female mice are shown starting from average number of genes across samples from each sex. The numbers of genes obtained after performing each step are indicated.

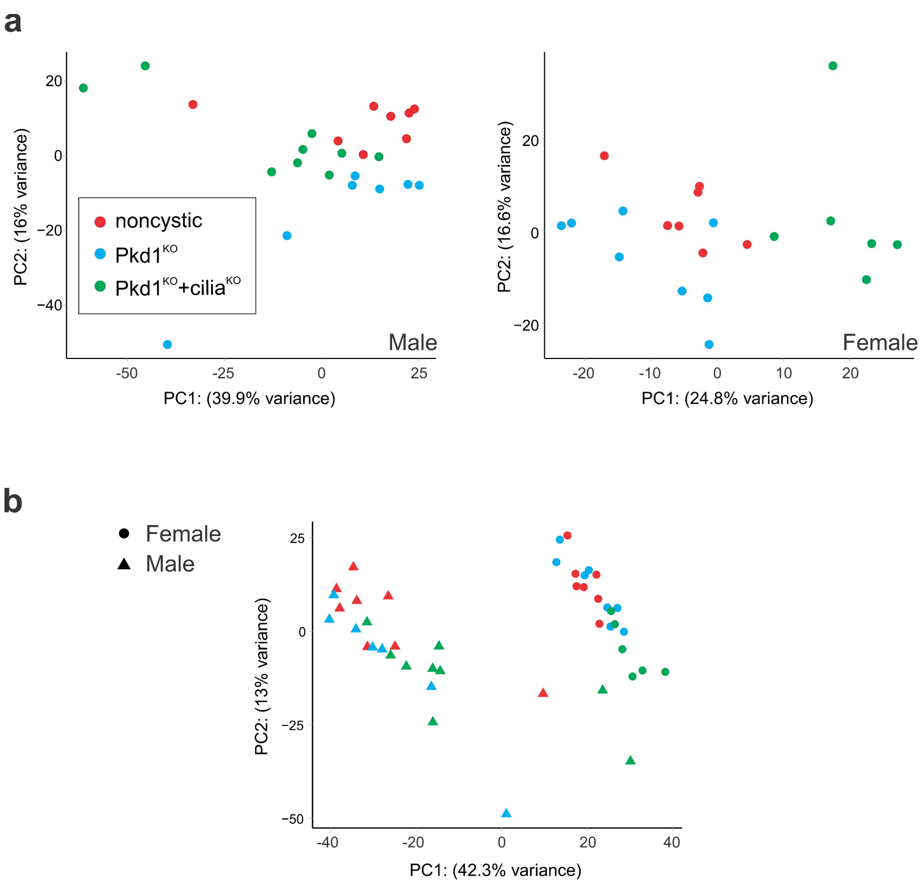

Supplementary Figure 3: PCA analysis for TRAP RNASeq. The percentages on each axis represent the percentages of variants explained by the respective principal components. **a,** PCA plot of male and female samples. Data points are colored by genotype group, and almost all samples have values close to their expected genotype groups. **b,** PCA plot showing sex differences of the TRAP RNASeq translatome profiles in 7-week kidneys.

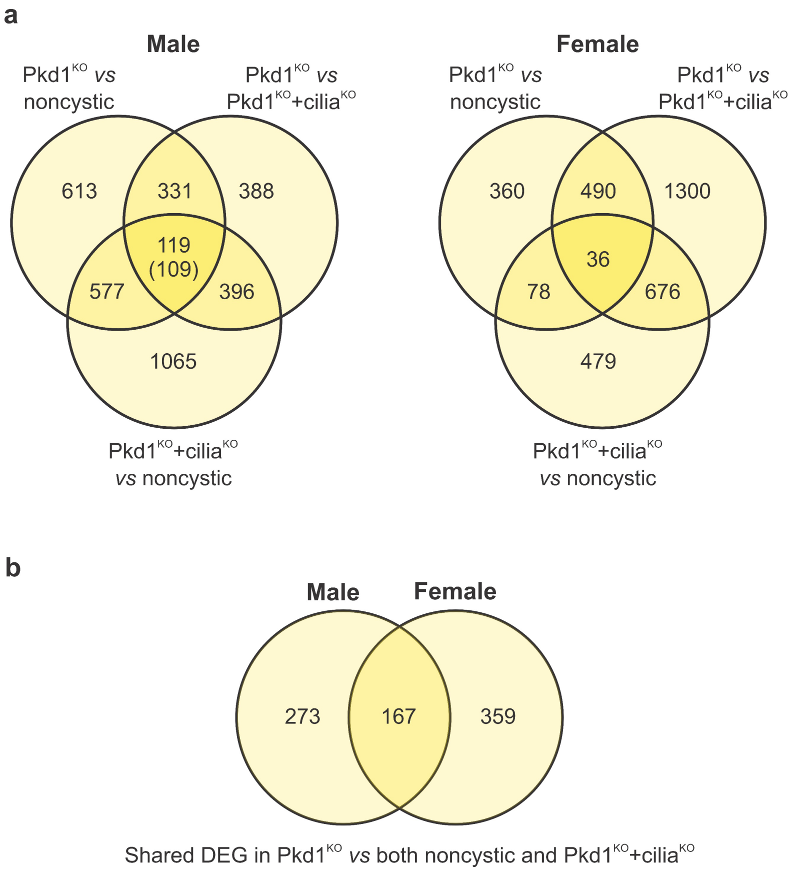

Supplementary Figure 4: Venn diagrams showing DEG distributions in TRAP RNASeq. a, Venn diagrams showing numbers of DEGs identified for each sex in the indicated pairwise analyses and in Pkd1^{KO} compared to both other groups. The number in parentheses (**a**) indicates Pkd1^{KO} DEGs changed in the same direction relative to the other two groups, when different from the total number of DEG. **b,** The DEGs in Pkd1^{KO} compared to both other groups and shared between the sexes (n=167).

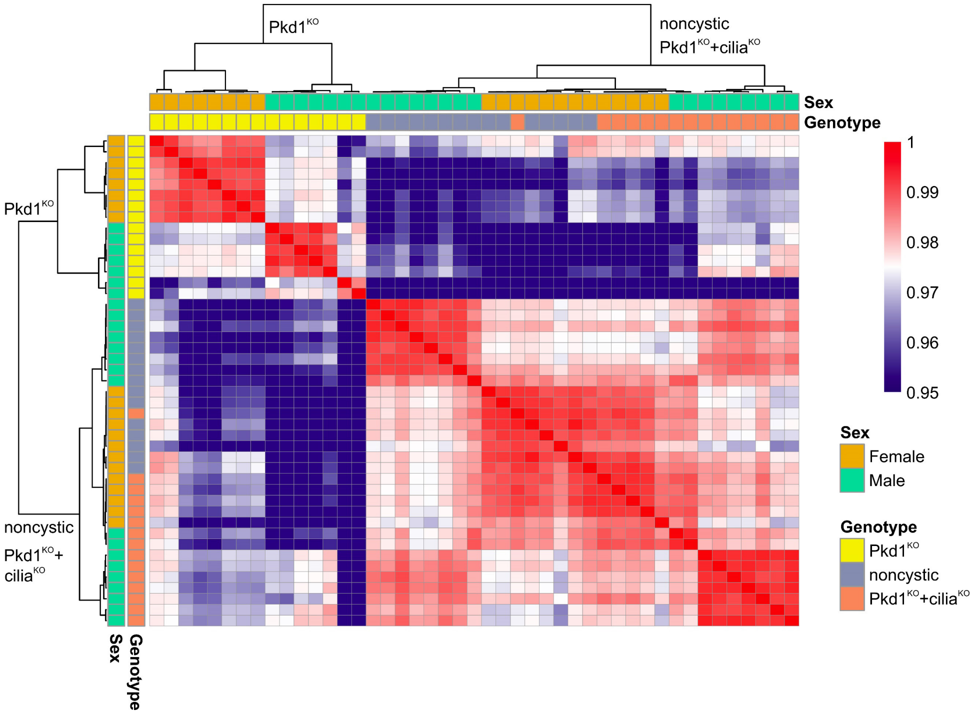

Supplementary Figure 5: Clustered heatmap of Pearson correlation coefficients. Unsupervised clustering of Pearson correlation coefficients based on TPM values for 799 unique DEGs in the combined male and female DEG sets comparing Pkd1^{KO} to Pkd1^{KO}+cilia^{KO} and noncystic kidneys. Pkd1^{KO} cluster together and there is further sub-clustering by sex within the groups.

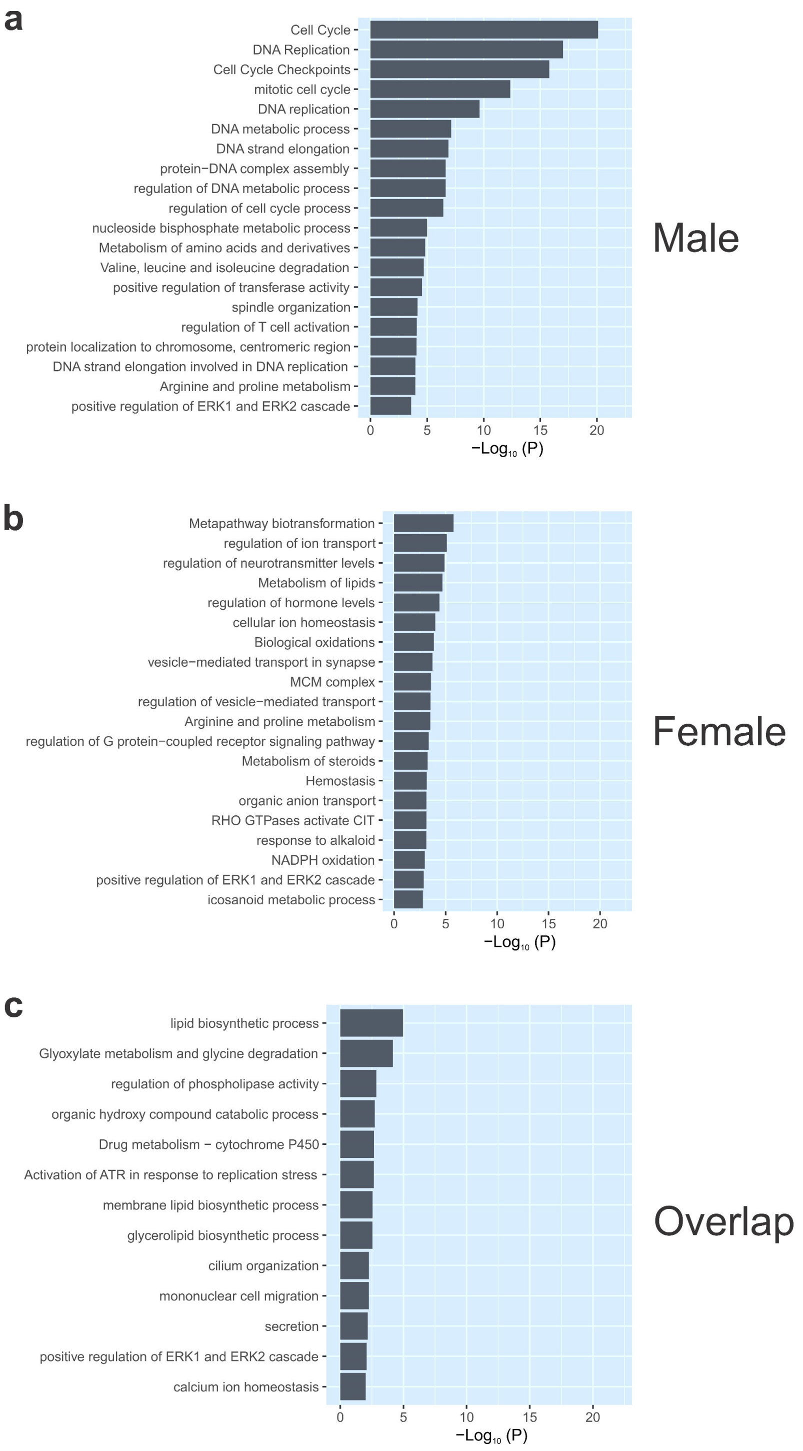

Supplementary Figure 6: Functional enrichment analysis of DEGs using Metascape. X-axis represents enrichment magnitude. **a,** The statistically significantly enriched set of 440 genes with same direction in Pkd1^{KO} compared to both noncystic and Pkd1^{KO}+cilia^{KO} in male mice. **b,** The statistically significantly enriched set of 526 genes with same direction in Pkd1^{KO} compared to both noncystic and Pkd1^{KO}+cilia^{KO} in female mice. **c,** The statistically significantly enriched set of 167 genes with same direction in Pkd1^{KO} compared to both noncystic and Pkd1^{KO}+cilia^{KO} in common between male and female mouse kidneys.

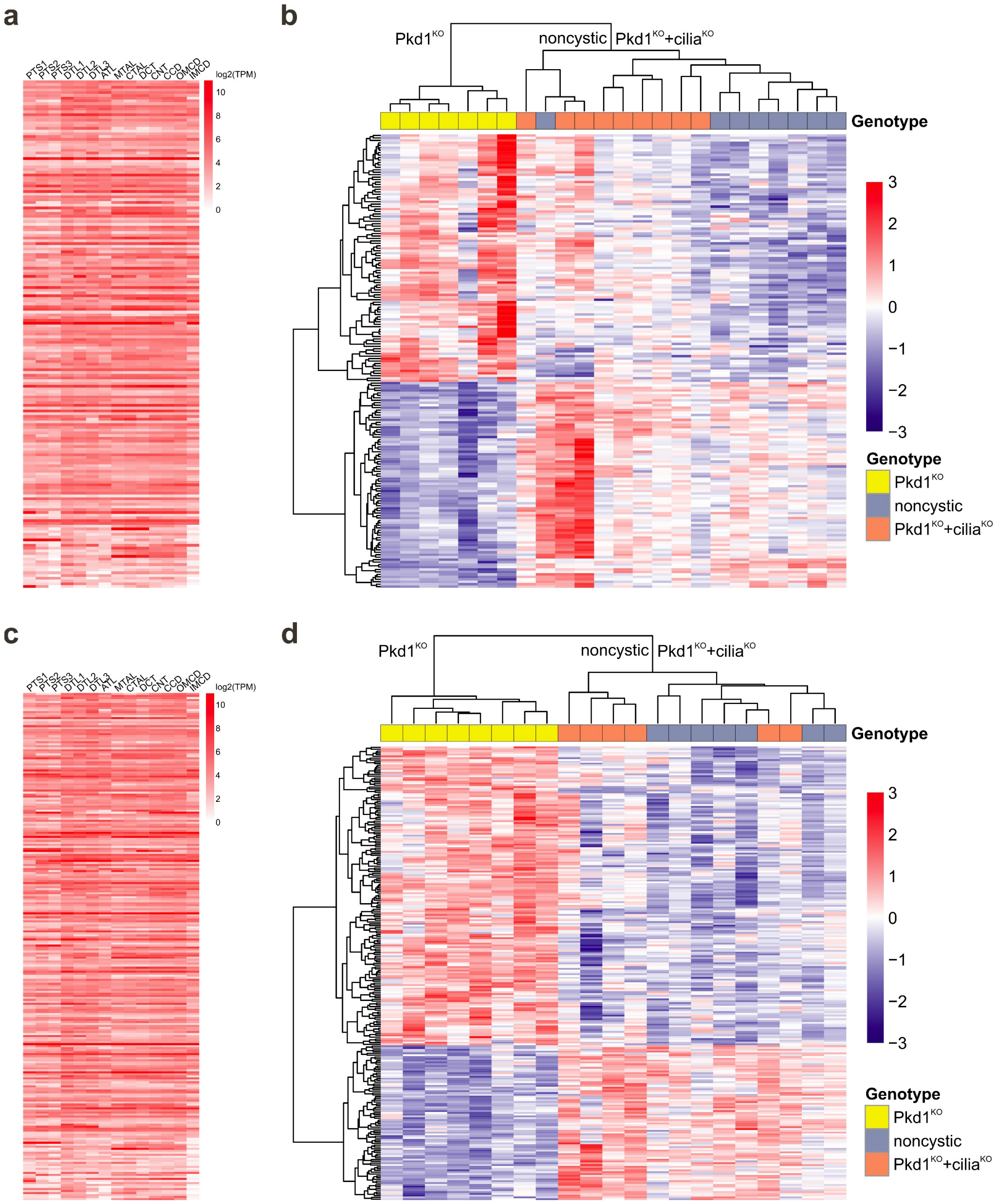

Supplementary Figure 7: Heatmaps showing expression of 'CDCA pattern' DEG. a,b, Data for male mice and **c,d** data for female mice. **a,c**, Heatmaps using published bulk RNASeq expression data from 14 microdissected normal nephron segments showing the subset of (**a**) 185 out of 440 and (**c**) 252 out of 526 of the CDCA pattern DEG with TPM >1.0 across most of the kidney tubule for male and female mice, respectively. The heatmaps present bulk RNASeq data from Supplementary Table 4 which also provides the key to identification the abbreviations for the 14 nephron segments. **b,d**, Heatmaps showing hierarchical clustering TRAP RNASeq expression data for the **a** and **c**, respectively. These are the 185 male and 252 female genes that showed significant same relative direction change in expression in Pkd1^{KO} compared to noncystic and Pkd1^{KO}+cilia^{KO} in both male and female mice with expression TPM >1 reported in a majority of microdissected kidney tubule segments in normal kidney.

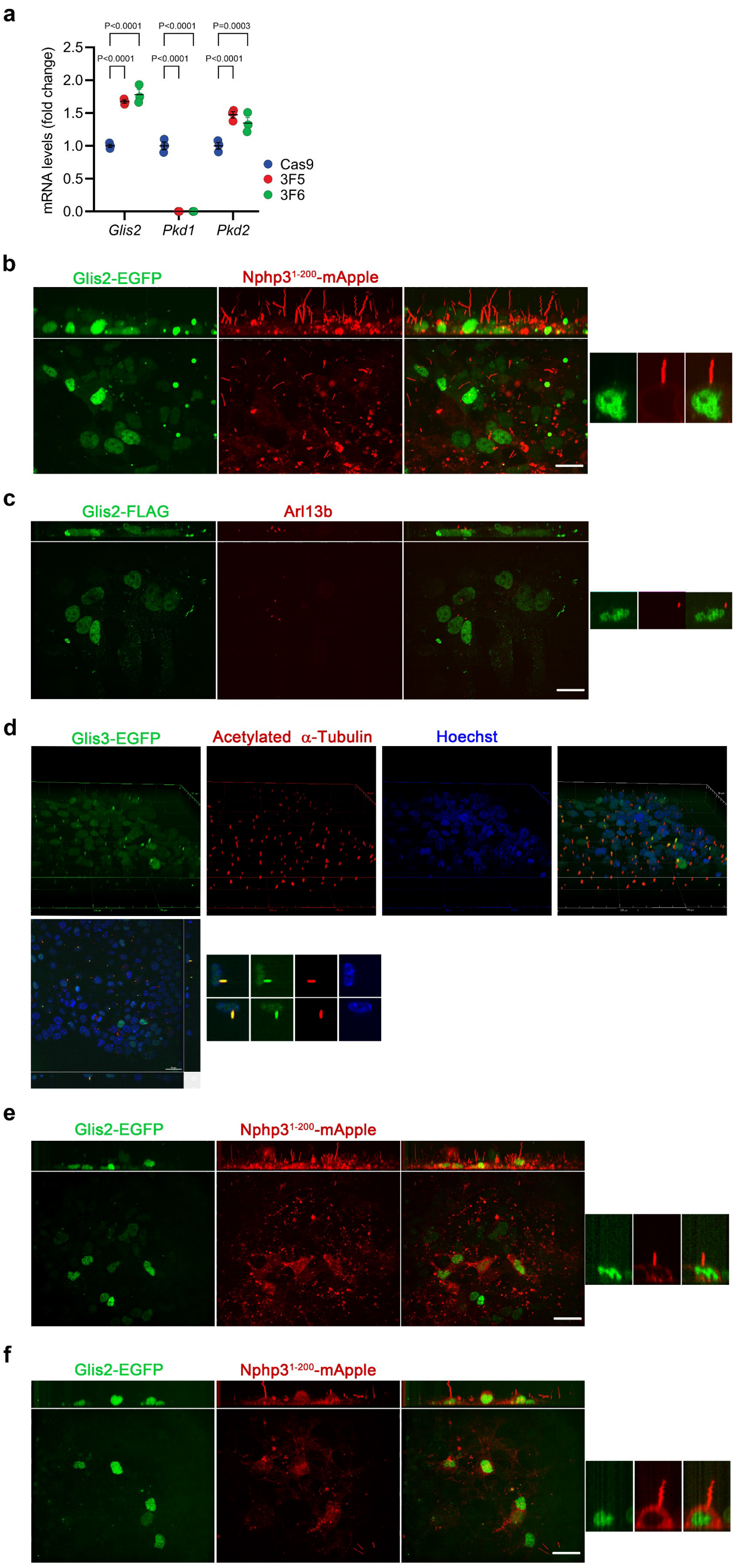

Supplementary Figure 8: Glis2 is undetectable in primary cilia. a, mRNA expression of *Glis2*, *Pkd1* and *Pkd2* in IMCD3 cells with *Pkd1* knockout (clones 3F5 and 3F6) compared to the parental control cell line (Cas9). Fold-change is shown relative to the mean of the ratio in the control cell line, which is set to 1.0. n=3 for each condition. Multiple-group comparisons were performed using two-way ANOVA followed by Dunnett's multiple-

comparison test, presented as the mean ± s.e.m. **b,** Representative images from wildtype IMCD3 cells expressing C-terminal Glis2-EGFP (green) and the cilia marker protein Nphp3$^{1\text{-}200}$-mApple (red) under live cell imaging conditions. Left three panels are maximum intensity projections. Right three panels are a single plane from a z-stack. Glis2-EGFP epifluorescence is seen in the nucleus but is absence from cilia marked by Nphp3$^{1\text{-}200}$-mApple. **c,** Representative images from transiently transfected HEK-293T cells expressing C-terminal Glis2-FLAG (green) with immunostaining of anti-FLAG and the cilia marker anti-Arl13b (red). Left three panels are maximum intensity projections. Right three panels are a single plane from a z-stack. **d,** Representative images of IMCD3 cells expressing C-terminal Glis3-EGFP (green) epifluorescence and immunostaining with acetylated α-tubulin (red). Nuclei are stained in blue with Hoechst. Glis3-EGFP epifluorescence is readily detectable in cilia. **e,** Representative images of IMCD3-Cas9 control cells expressing Glis2-EGFP (green) and Nphp3$^{1\text{-}200}$-mApple (red) under live cell imaging conditions. Left three panels are maximum intensity projections. Right three panels are a single plane from a z-stack. **f,** Representative images of IMCD3–3F6 Pkd1^{KO} cell line expressing Glis2-EGFP (green) and Nphp3$^{1\text{-}200}$-mApple (red) under live cell imaging conditions. Left three panels are maximum intensity projections. Right three panels are a single plane from a z-stack. Glis2-EGFP epifluorescence is seen in the nucleus in both **e**,**f**, but is absent from cilia marked by Nphp3$^{1\text{-}200}$-mApple. All experiments were done in cell lines transduced with lentiviral vectors; all experiments were repeated at least 3 times and showed consistent results for the representative images shown. Scale bars, 25 µm. Source data for exact values are provided as a Source Data file.

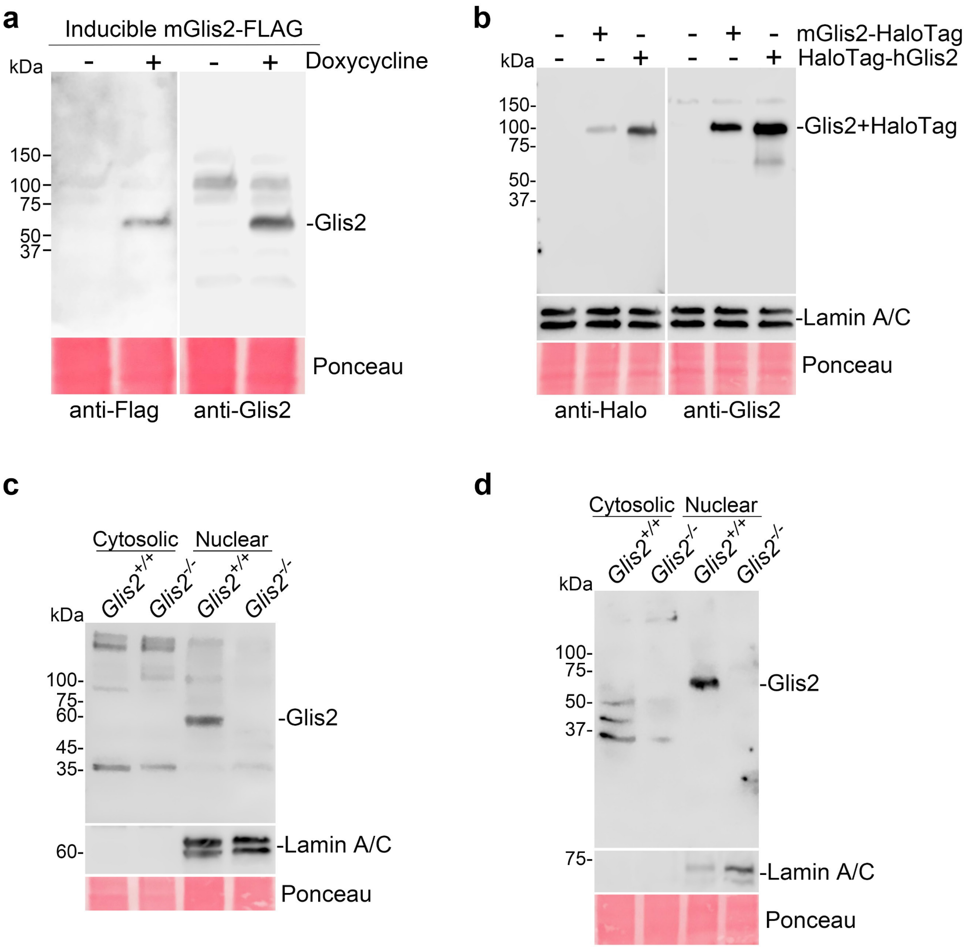

Supplementary Figure 9: The polyclonal anti-Glis2 antibody YNG2 detects mouse and human Glis2. a, Total cell lysates of HEK-293T cells stably expressing doxycycline inducible C-terminal mouse Glis2-FLAG (mGlis2-FLAG) were immunoblotted with anti-FLAG antibodies and anti-Glis2 (YNG2) antibodies without (-) and with (+) doxycycline treatment. Ponceau staining is shown as loading control. **b,** Nuclear lysates of HEK-293T cells transfected with C-terminal mouse Glis2-HaloTag (mGlis2-Halo) or N-terminal human HaloTag–Glis2 (Halo-hGLIS2) were immunoblotted with anti-Halo and anti-Glis2 (YNG2) antibodies. Lamin A/C is shown for nuclear loading. Ponceau staining is shown for total loading control. **c,** SV40 transformed cell lines were made from primary cell cultures of kidneys of *Glis2*$^{-/-}$ null and *Glis2*$^{+/+}$ wild type mice and lysates of cytosolic and nuclear fractions were obtained from each cell line. YNG2 detects a band migrating as expected for Glis2 only in the nuclear fraction of the wild type cell line. Lamin A/C is shown for nuclear enrichment and loading control. Total loading was controlled by Ponceau staining. **d,** Lysates of cytosolic and nuclear fractions from kidney tissue of *Glis2*$^{-/-}$ (*Glis2*$^{fl/fl}$*;Actb*^{Cre}) null and *Glis2*$^{+/+}$ wild type mice were immunoblotted with YNG2 antibodies. Lamin A/C is shown as nuclear enrichment and loading control. Glis2 is specifically detected only in lysates of the nuclear fraction of wild type kidney. Ponceau staining is shown for total loading. Full-length blots are provided as source data. Experiments in **a,d** were done at least three times; **b,c** were done two times. Source data for uncropped blots are provided as a Source Data file.

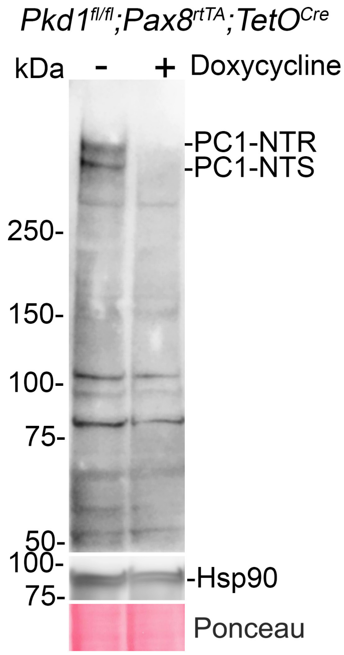

Supplementary Figure 10: Depletion of PC1 in primary cells from kidneys of $Pkd1^{fl/fl}$;$Pax8^{rtTA}$,$TetO^{Cre}$ mice after doxycycline treatment. Immunoblot showing the Endo H resistant (PC1-NTR) and Endo H sensitive (PC1-NTS) N-terminal fragments of PC1 detected by anti-PC1 clone 7e12 antibody in primary cells with indicated genotype without or with doxycycline treatment for 3 days. Doxycycline induced knockout results in absence of PC1. Hsp90 and Ponceau staining are shown as loading controls. Full-length blots are provided as source data. Experiment was done two times. Source data for images of uncropped blots are provided as a Source Data file.

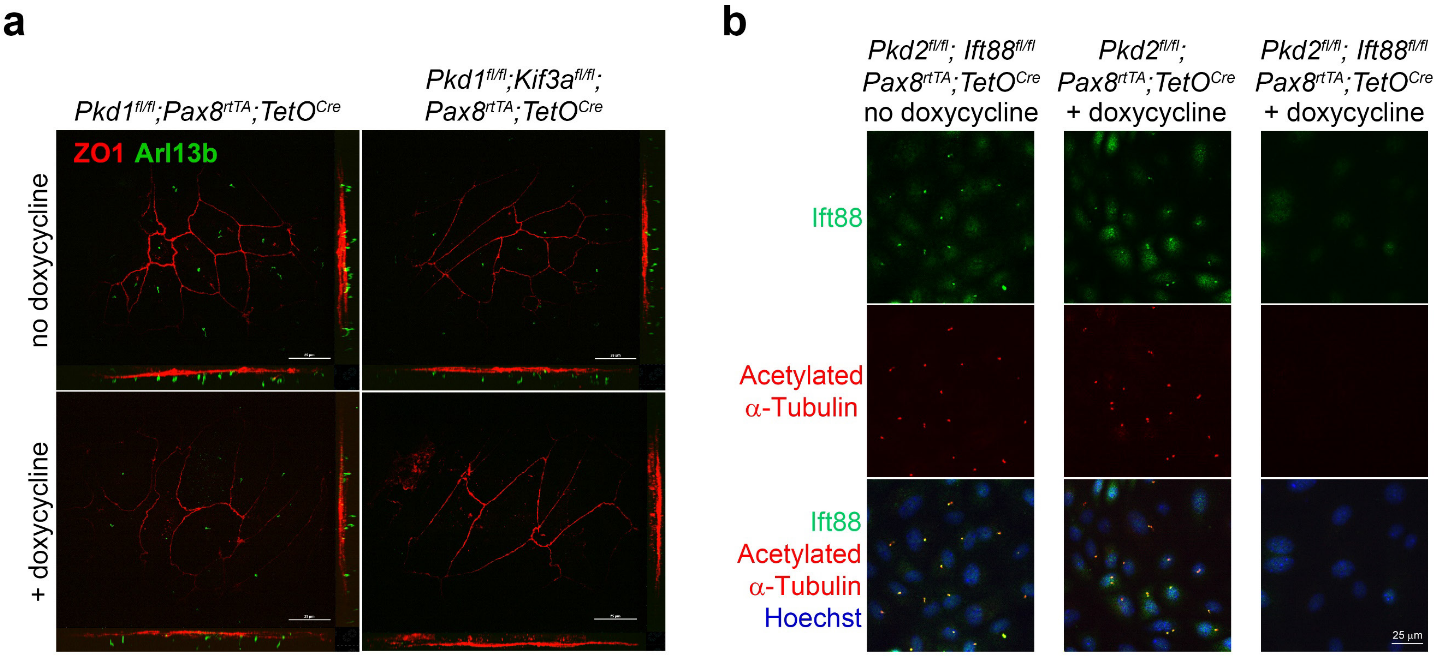

Supplementary Figure 11: Doxycycline treatment results in absence of cilia in Pkd1^{KO}+cilia^{KO} and Pkd2^{KO}+cilia^{KO} primary cells. a, Representative images of primary cells cultured from kidneys of mice with the indicated genotypes without or with doxycycline treatment for 3 days. Cells were fixed after 1 day serum starvation at the end of 20 days in culture cells were stained for ZO1 (red) to mark tight junctions and Arl13b (green) to mark cilia. Cilia are absent in double knockout cells after doxycycline treatment. **b,** Representative images of primary cells cultured from kidneys of mice with the indicated genotypes without or with doxycycline treatment for 3 days. Cells were serum starved for one day and stained for the cilia markers Ift88 (green) and acetylated α-tubulin (red) and the nuclear Hoechst stain (blue). Scale bars, 25 μm.

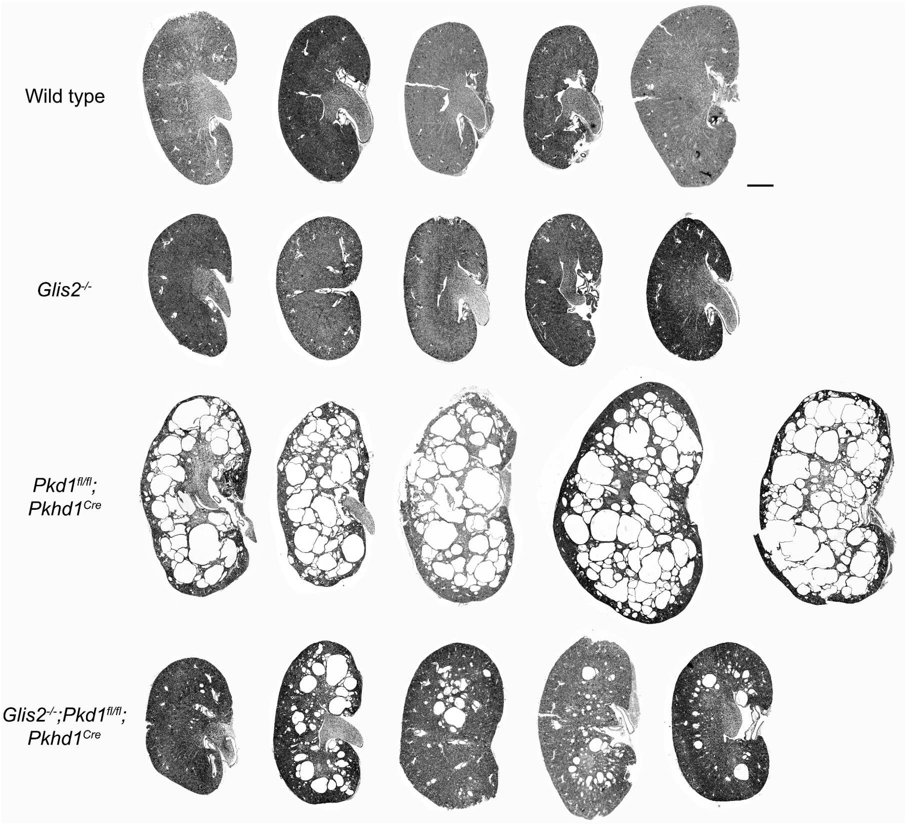

Supplementary Figure 12: Images of all the kidney histological sections used in Figure 3a-d. Scale bar, 1 mm.

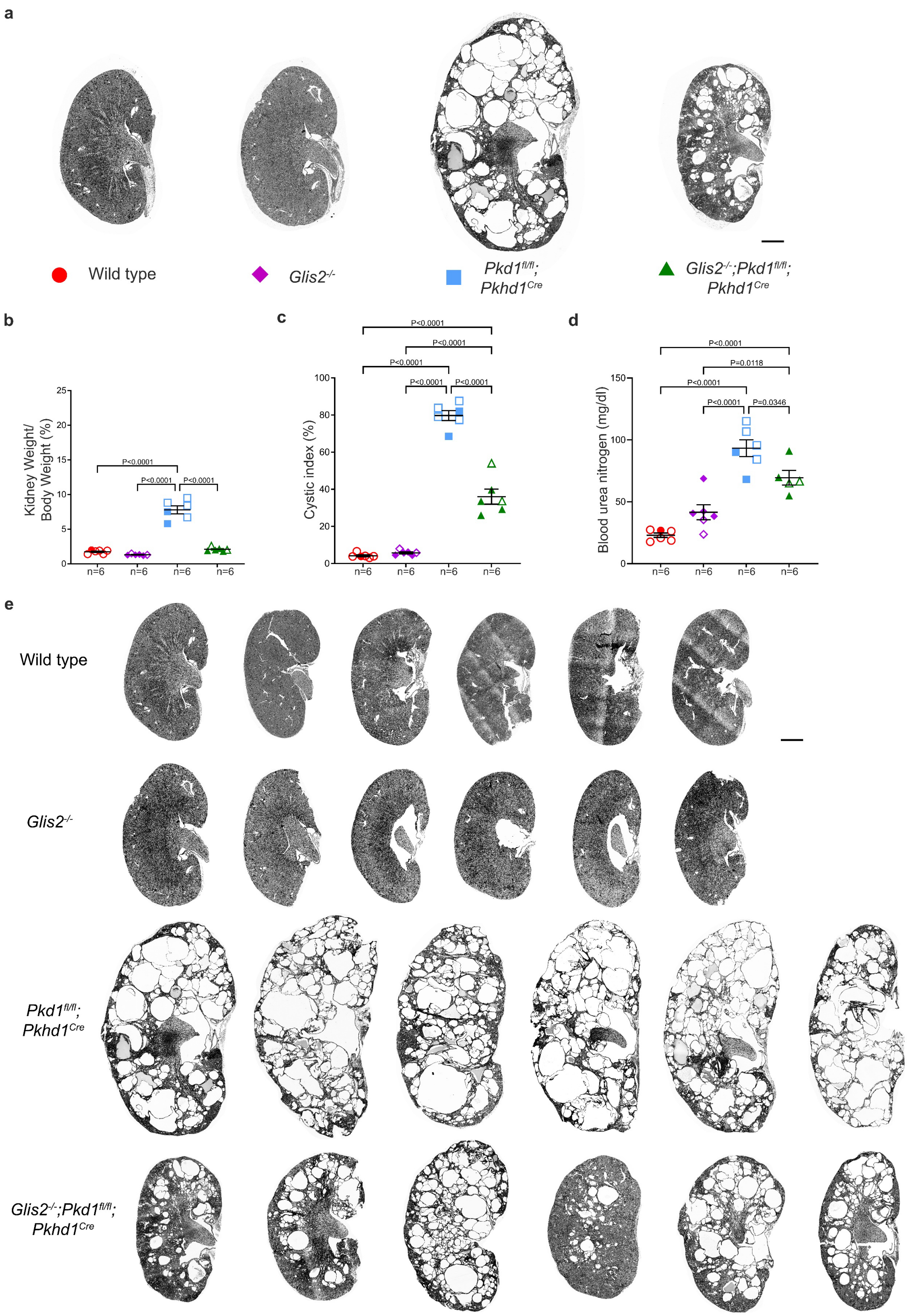

Supplementary Figure 13. *Glis2* inactivation shows sustained suppression polycystic kidney disease in an early onset mouse model. a, Representative images of kidneys with the indicated genotypes at P49. Scale bar, 1 mm. **b-d**, Aggregate quantitative data for the indicated parameters. *n*, number of mice in each group. Colors and symbol shapes correspond to genotypes defined in **a**. **e,** All kidney histological sections used in **a-d**. **b-d,** Male mice, closed symbols; female mice, open symbols. Multiple-group comparisons by one-way ANOVA followed by Tukey's multiple-comparison test, presented as mean±s.e.m. **e,** Scale bar, 1 mm. Source data for exact values are provided as a Source Data file.

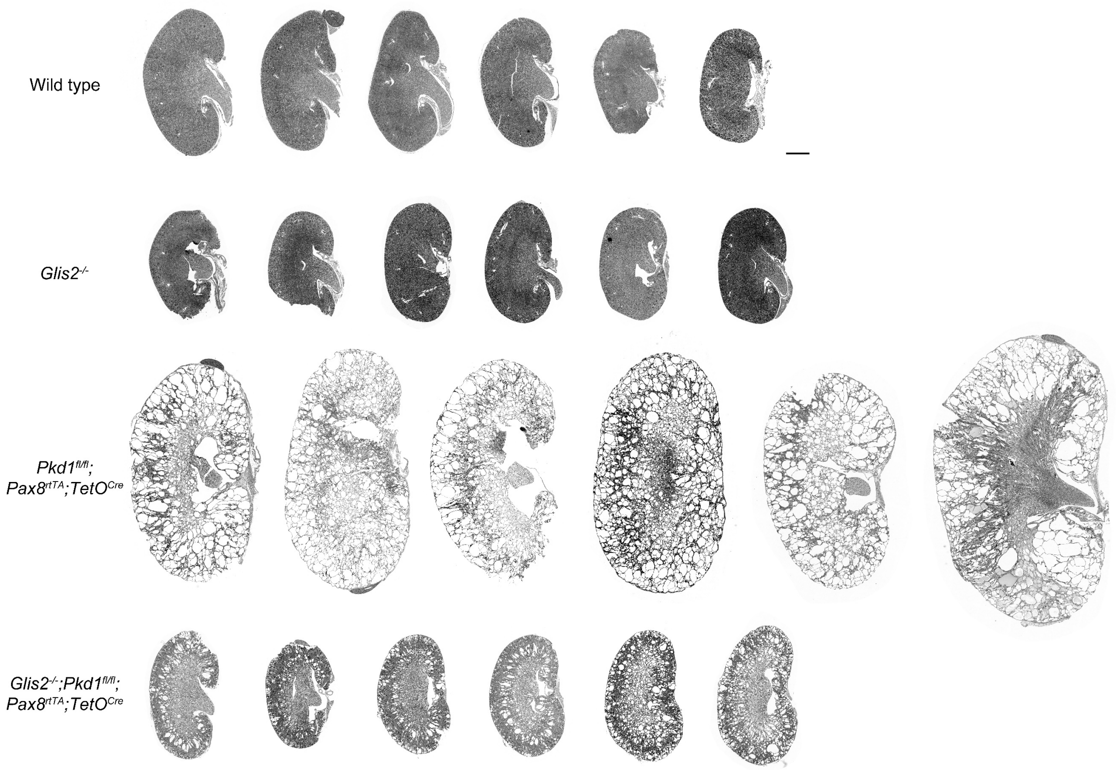

Supplementary Figure 14: Images of all the kidney histological sections used in Figure 3e-h. Scale bar, 1 mm.

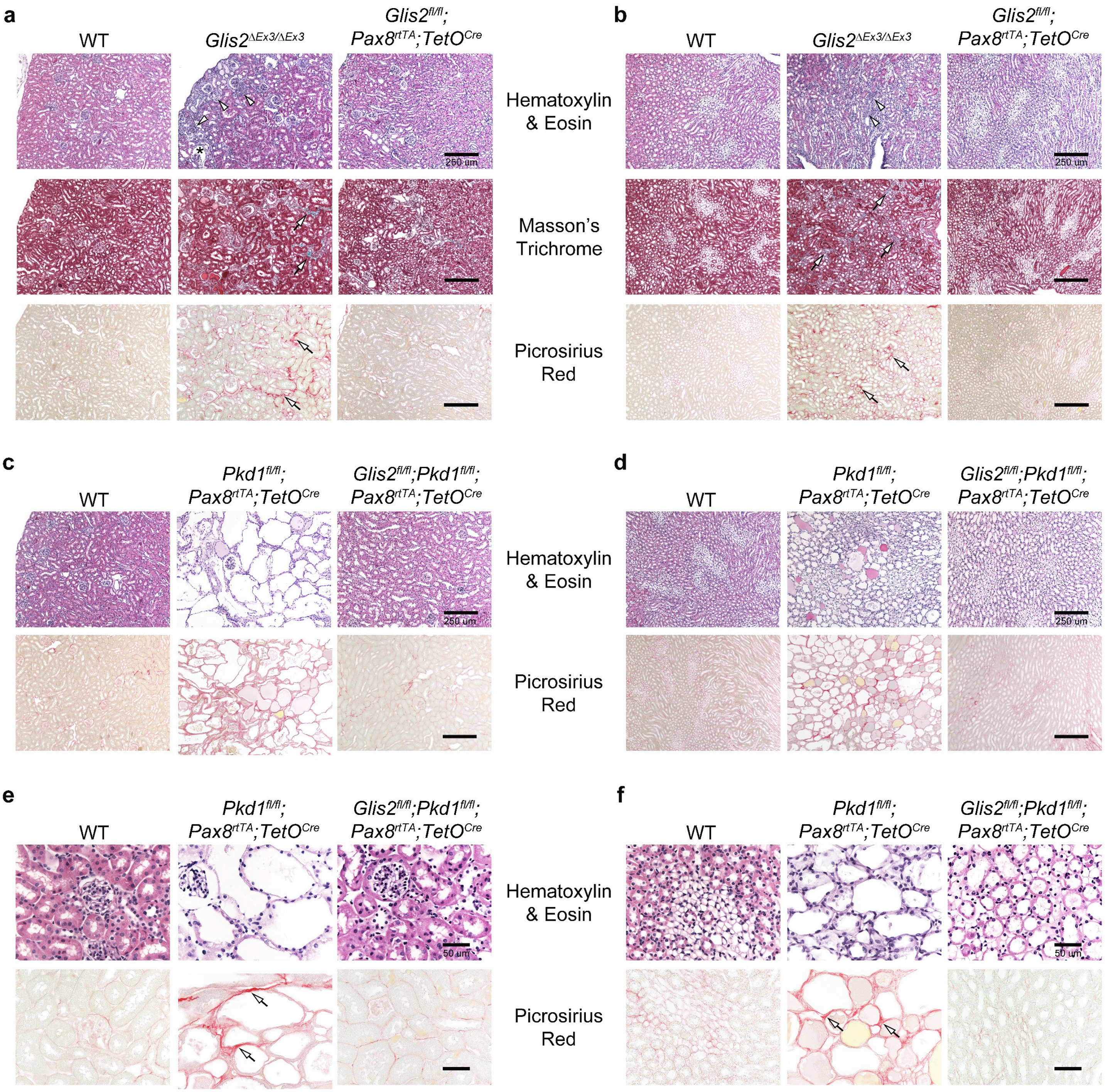

Supplementary Figure 15: Adult inactivation of *Glis2* does not result in a nephronophthisis-like phenotype. a-f, Representative images of kidney cortex (**a,c,e**) and medulla (**b,d,f**) from mice with the indicated genotypes at 18 weeks age. All mice with the *Pax8*^{rtTA}*;TetO*^{Cre} alleles received oral doxycycline to induce gene knockouts from P28-42. **a,b** Comparison of germline null *Glis2* $^{\Delta Ex3/\Delta Ex3}$ and adult inducible *Glis2* inactivation. *Glis2* $^{\Delta Ex3/\Delta Ex3}$ kidneys show inflammatory infiltrates (arrowhead, H&E) and interstitial fibrosis (arrows, blue areas in Masson's Trichrome and red areas in Picrosirius Red) in both cortex (**a**) and medulla (**b**). These changes are absent in tubule-selective adult conditional inactivation of *Glis2* (*Glis*$^{fl/fl}$*;Pax8*^{rtTA}*;TetO*^{Cre}). **c-f**, Fibrosis is present in the pericystic areas in the cortex (**c,e**) and medulla (**d,f**) of *Pkd1*$^{fl/fl}$*;Pas8*^{rtTA}*;TetO*^{Cre} kidneys (arrows in **e,f**) but

absent from the *Glsi2*$^{fl/fl}$*;Pkd1*$^{fl/fl}$*;Pas8*^{rtTA}*;TetO*^{Cre} double knockout kidneys which are protected from cyst formation. Scale bars: 250 µm (**a-d**); 50 µm (**e, f**).

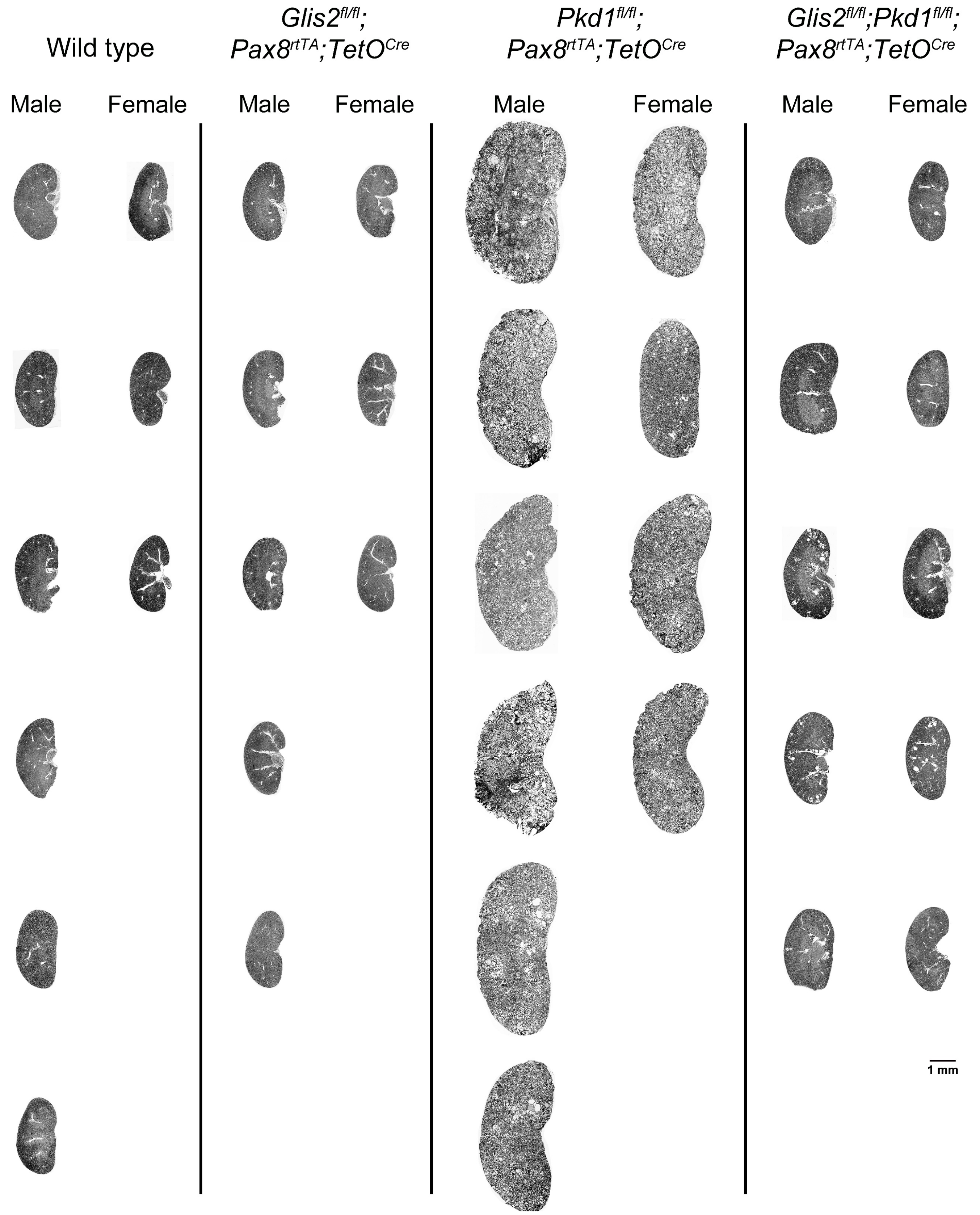

Supplementary Figure 16: Images of all the kidney histological sections used in Figure 3i-l. Genotypes and sexes for each kidney are shown at the top. Scale bar, 1 mm

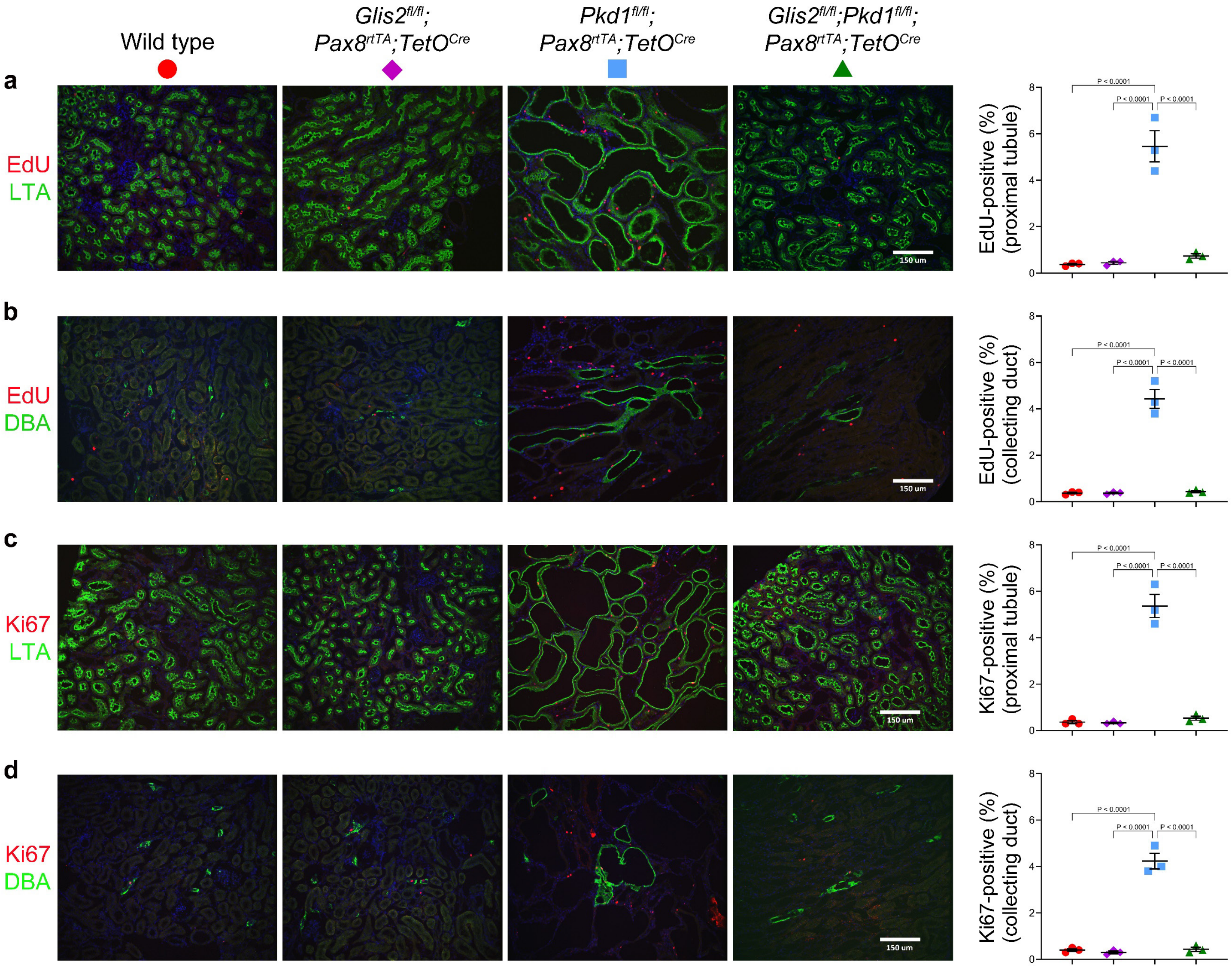

Supplementary Figure 17: Concomitant inactivation of *Glis2* decreases cyst cell proliferation in adult onset ADPKD. a-d, Representative images and aggregate quantitative data showing the percentage of EdU-positive nuclei (**a, b**) and Ki67-positive nuclei (**c, d**) in Lotus tetragonolobus agglutinin (LTA) positive proximal tubules (**a,c**) and Dolichos biflorus agglutinin (DBA) positive collecting ducts (**b,d**) in kidneys of mice with the indicated genotypes at 18 weeks. All mice received doxycycline from P28–42. The percentage of EdU and Ki67 positive nuclei was determined by counting at least 1,000 LTA or DBA positive nuclei marked by Hoechst 33342 (blue) in each mouse (n=3 mice). Multiple-group comparisons were performed by one-way ANOVA followed by Tukey's multiple-comparison test and data are presented as mean±s.e.m. Scale bars, 150 µm. Source data for exact values are provided as a Source Data file.

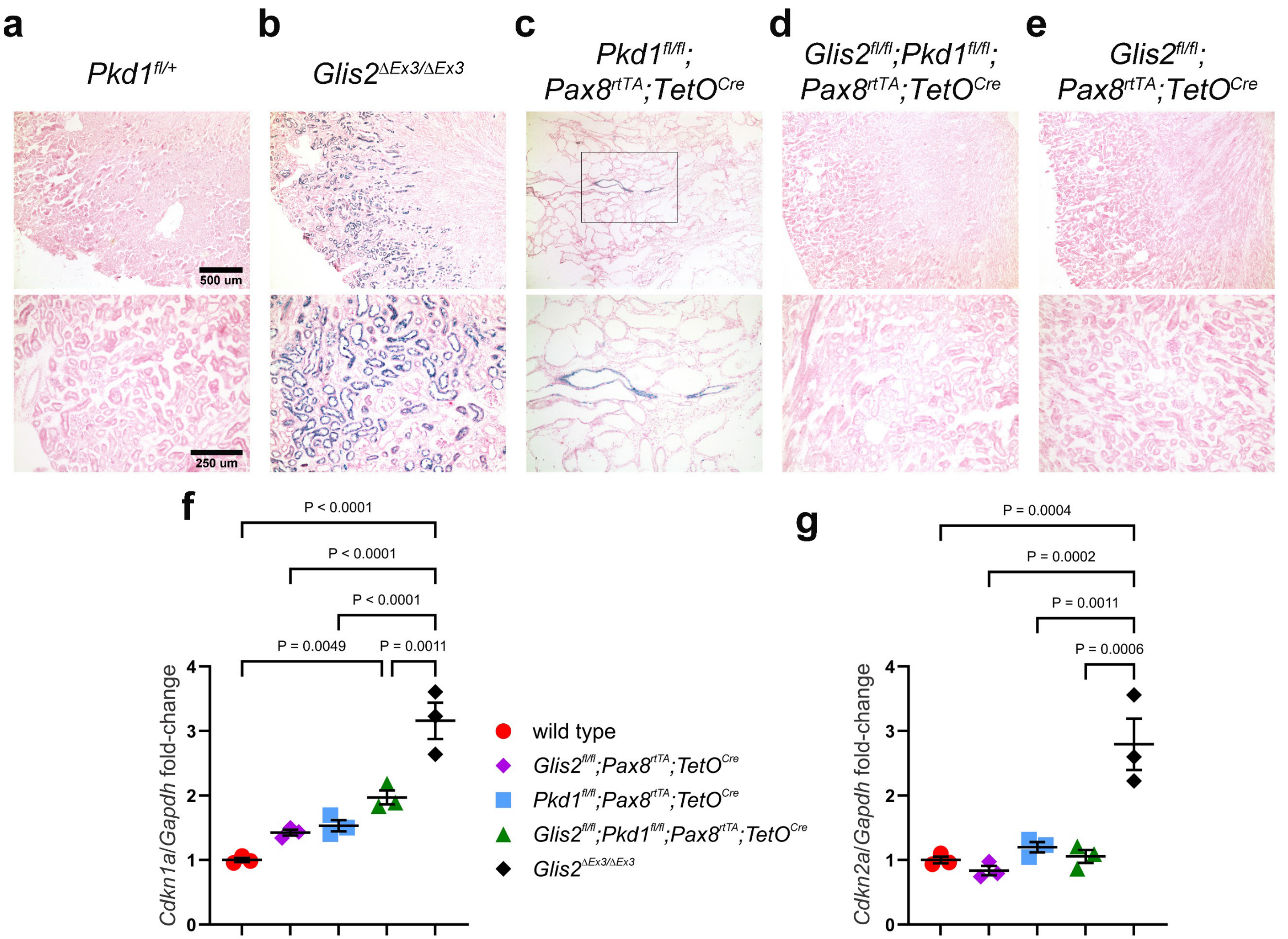

Supplementary Figure 18: Inducible inactivation of *Glis2* does not result in senescence in kidney epithelial cells. a-e, Representative bright-field microscopy images of kidney sections showing senescence-associated β-galactosidase (SA-β-Gal) activity with the specified genotypes at 18 weeks with lower (*upper panel*) and higher (*lower panel*) magnification. Blue staining indicative of cellular senescence is present in the cortical region of germline null *Glis2* $^{\Delta Ex3/\Delta Ex3}$ kidneys (**b**) but is absent from kidneys of *Glis2*$^{fl/fl}$; *Pkd1*$^{fl/fl}$; *Pax8*^{rtTA}; *TetO*^{Cre} and *Glis2*$^{fl/fl}$; *Pax8*^{rtTA}; *TetO*^{Cre} kidneys (**d,e**). Occasional cysts showing evidence of cellular senescence are observed in cystic *Pkd1*$^{fl/fl}$; *Pax8*^{rtTA}; *TetO*^{Cre} kidneys (**c**; boxed region is shown at higher magnification in lower panel). Histological sections from two mice for each genotype were examined to identify representative images. Scale bars: 500 μm (upper panel); 250 μm (lower panel). **f,g**, qRT-PCR *Cdkn1a* mRNA (**f**) and *Cdkn2a* mRNA (**g**) expression in whole kidney lysates from mice with the indicate genotypes at 18 week of age, normalized to *Gapdh* and expressed as fold-change relative to the mean for wild type kidneys which is set to 1.0. All mice except *Glis2*$^{\Delta ex3/\Delta ex3}$ (null) received oral doxycycline from P28-42. *n*=3 mice for each group. Multiple-group comparisons are done by one-way ANOVA followed by Tukey's multiple-comparison test and are presented as the mean± s.e.m. Source data for exact values are provided as a Source Data file.

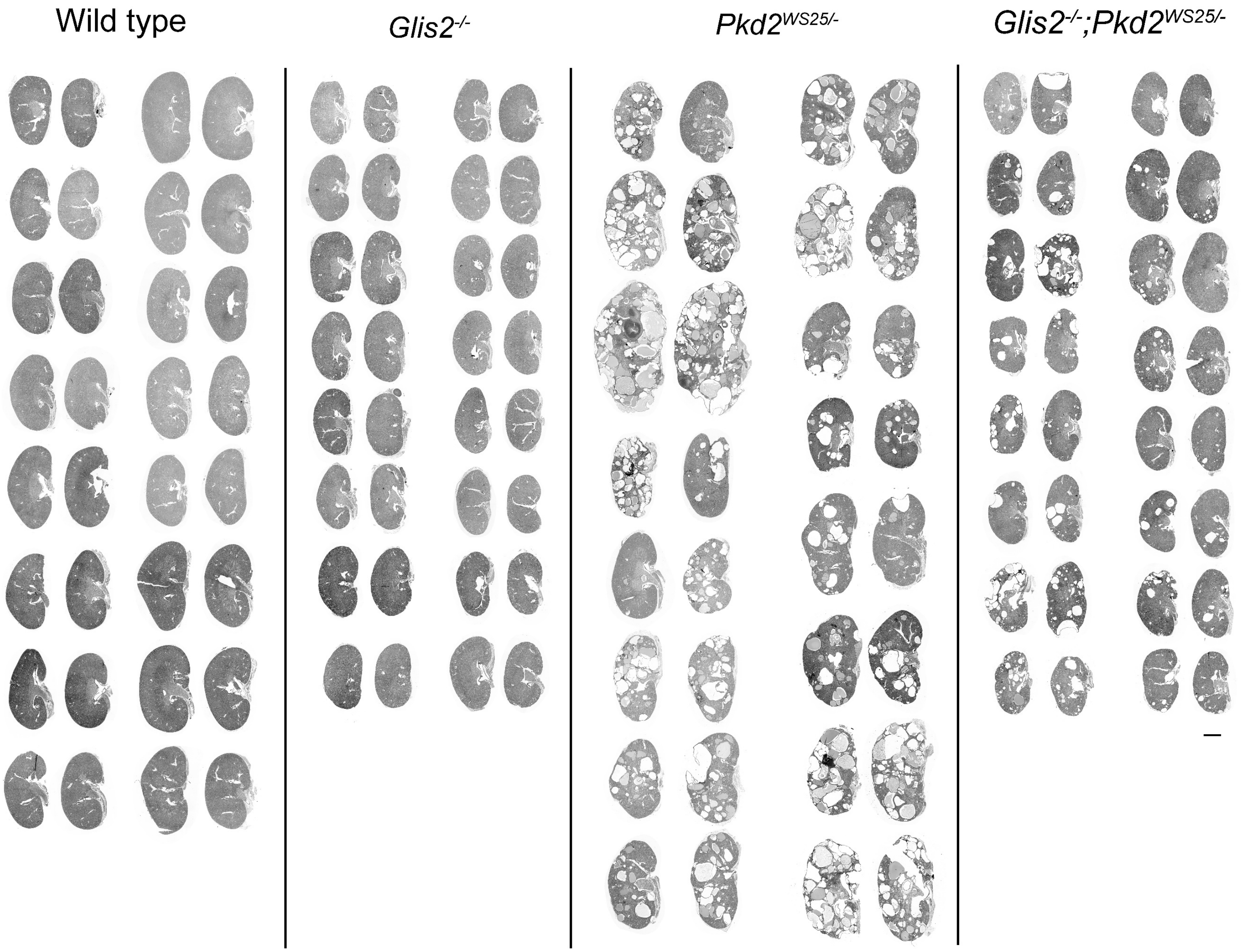

Supplementary Figure 19: Images of all the kidney histological sections used in Figure 3m-p. Pairs of kidneys from each mouse are shown and each kidney is used as a separate data point in the cystic index data in Figure 3m due to the variation in cyst burden between two kidneys from the same mouse in the *Pkd2*$^{WS25/-}$ models. Scale bar, 1 mm

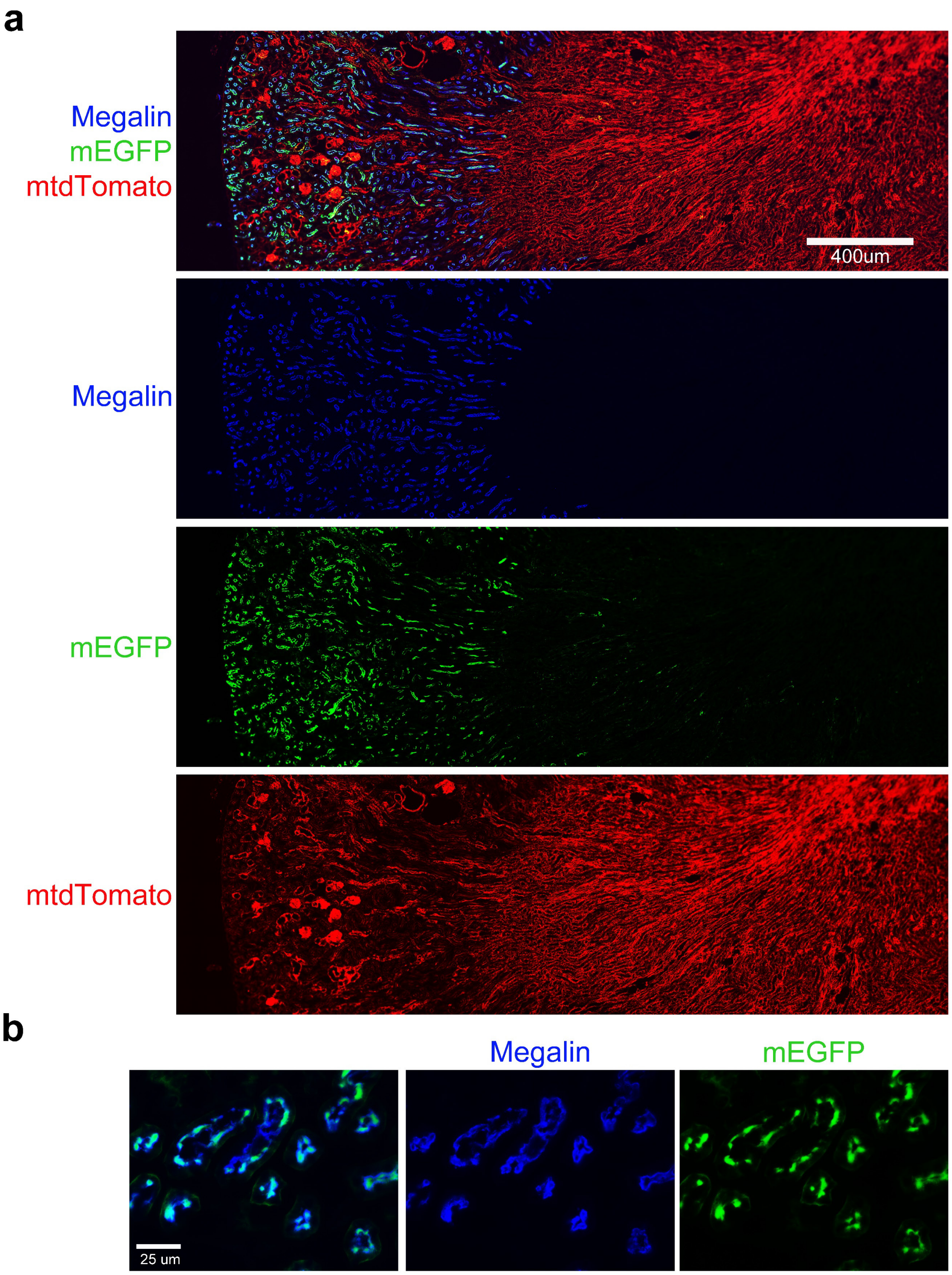

Supplementary Figure 20: *UBC$^{Cre/ERT2}$* is active only in proximal tubule in mouse kidneys. a,b Colocalization of Cre activity (mEGFP) with proximal tubule marker megalin. Mice with *UBC$^{Cre/ERT2}$* and the Cre reporter *ROSA$^{mT/mG}$* allele *(UBC$^{Cre/ERT2}$;ROSA$^{mT/mG}$*) received tamoxifen daily from P28-35 and kidney tissue was examined at P35. Segments with Cre activity are marked by mEGFP (green); absence of Cre activity is marked by mtdTomato (red). Proximal tubules are marked by anti-megalin antibody staining (blue). There is complete overlap between EGFP and anti-megalin indicating that *UBC$^{Cre/ERT2}$* is only active in the proximal tubule in the kidney. Image of representative of sections from 3 independent mice; mice aged past P35 show the same pattern (data not shown). Scale bars: 400 µm (**a**); 25 µm (**b**).

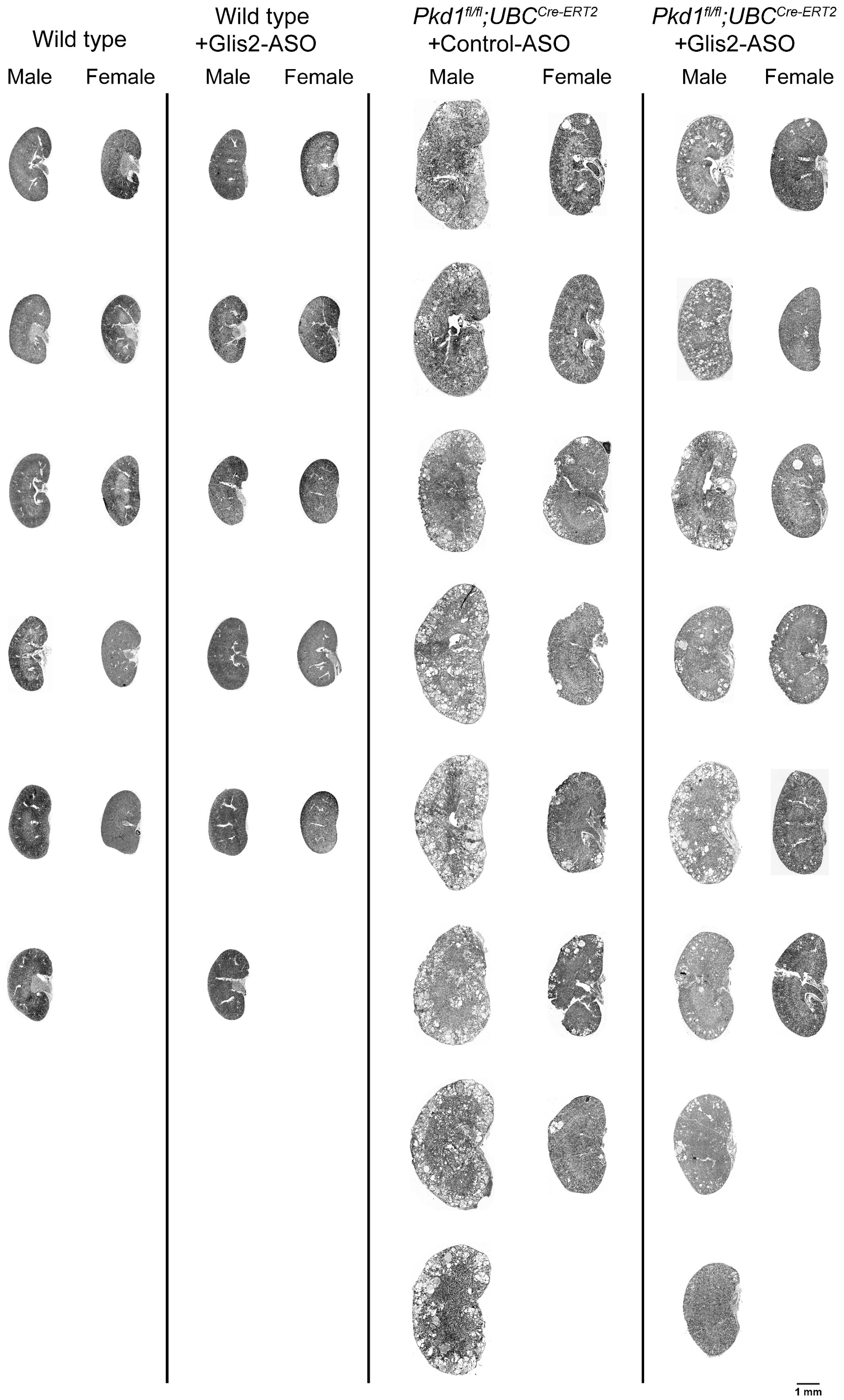

Supplementary Figure 21. Images of all the kidney histological sections used in Figure 4. Genotypes, treatments, and sexes for each kidney are shown at the top. Scale bar, 1 mm.

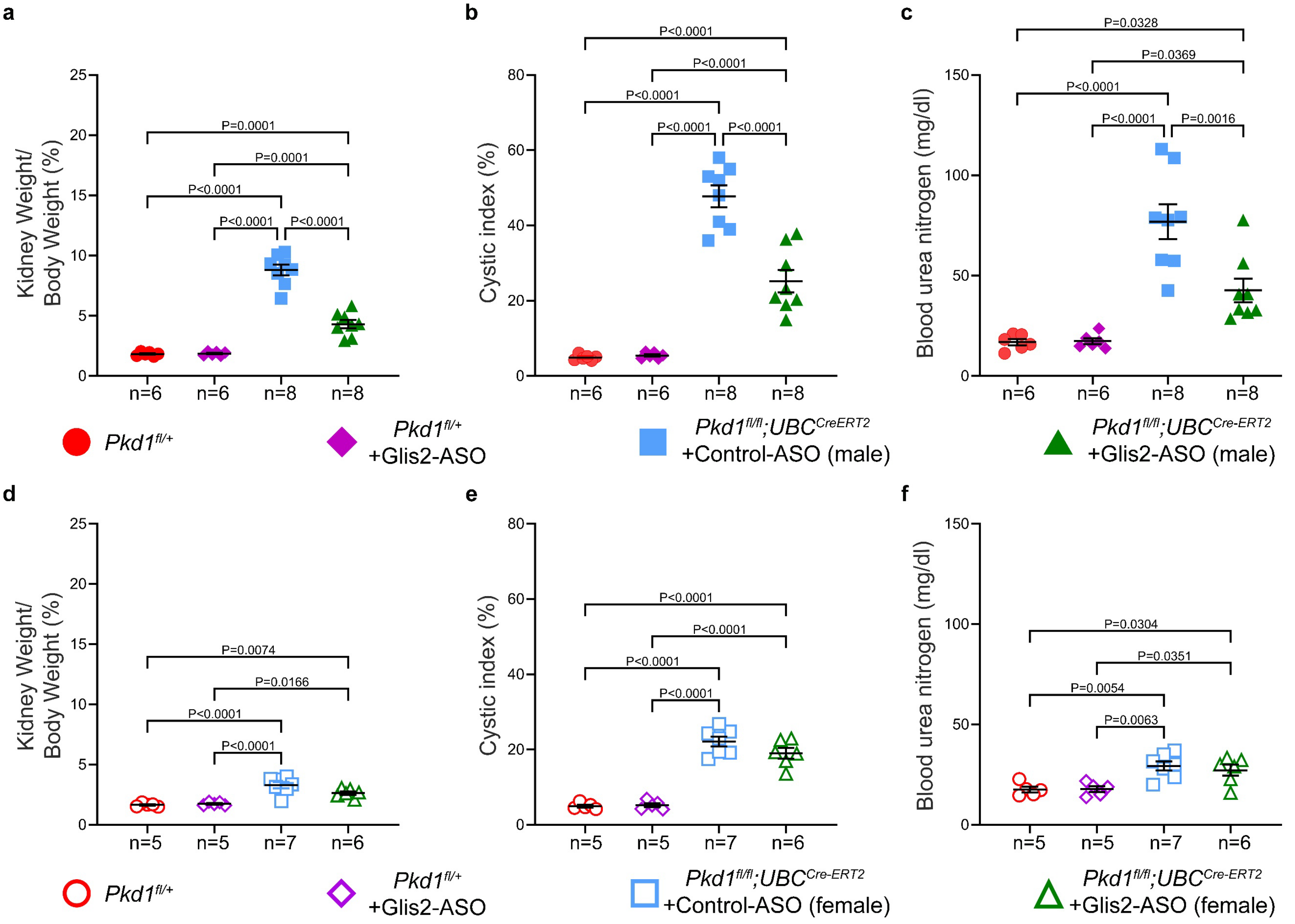

Supplementary Figure 22. Aggregate quantitative data in Fig. 4b-d separated by sex. a-f, Aggregate quantitative data for the indicated parameters in male (**a-c**) and female (**d-f**) mice. *n*, number of mice in each group. Colors and symbol shapes correspond to indicated genotypes and treatment groups. Multiple-group comparisons are done by one-way ANOVA followed by Tukey's multiple-comparison test presented as mean±s.e.m. Source data for exact values are provided as a Source Data file.

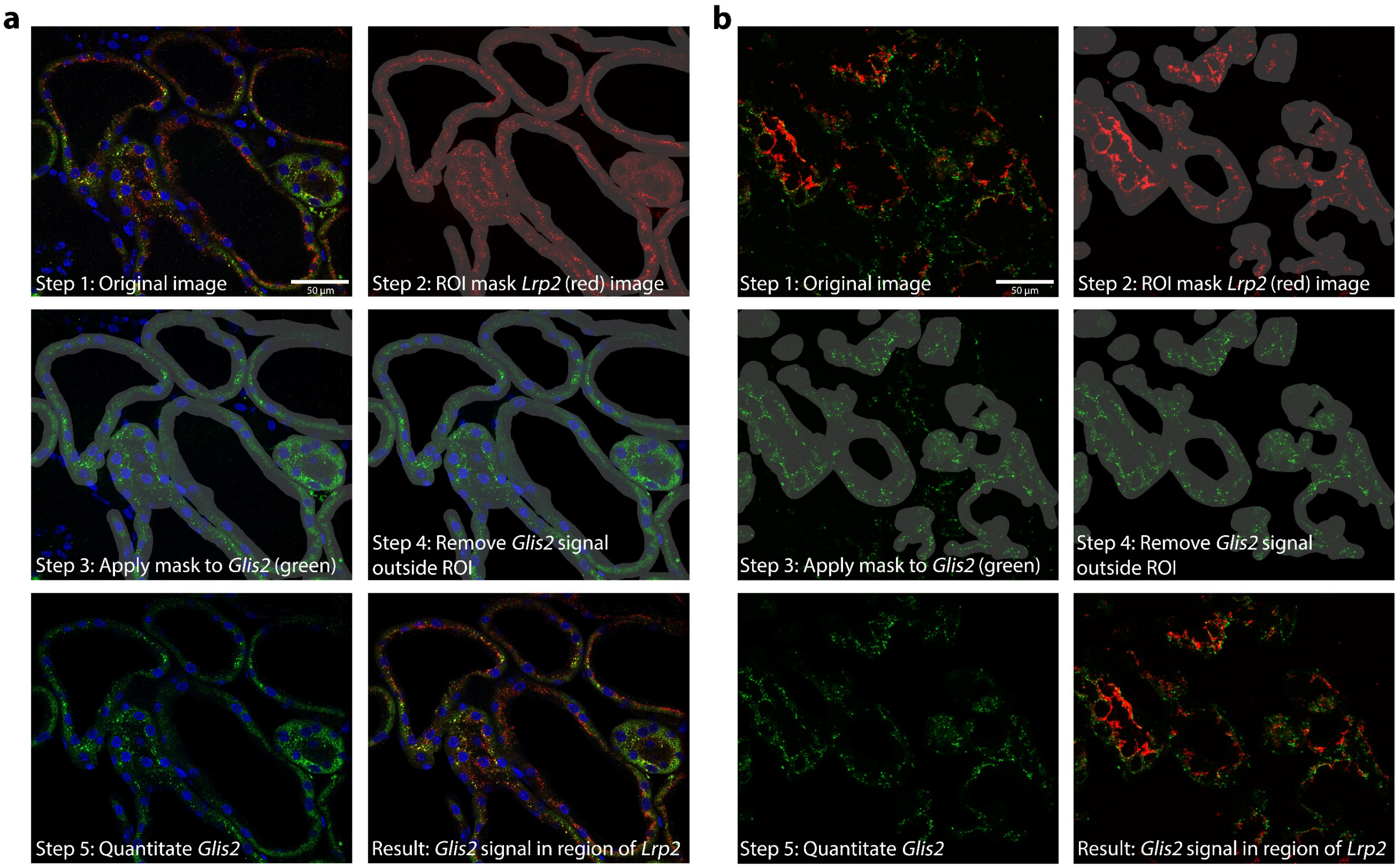

Supplementary Figure 23. Approach to quantifying *Glis2* mRNA expression in proximal tubules. a,b Representative images showing the workflow and key steps in masking strategies used for quantitation in both smFISH **(a)** and RNAScope-FISH (**b**). A mask showing the regions of interest (ROI) was created solely based on the megalin (*Lrp2*) channel (red). The resulting mask was applied to the *Glis2* channel (green). Signals in areas outside the ROI in the *Glis2* channel were removed. The *Glis2* signals remaining in the ROI were quantified with Cell Profiler. DAPI stained nuclei in the ROI were also counted by Cell Profiler. Scale bar, 50 µm.

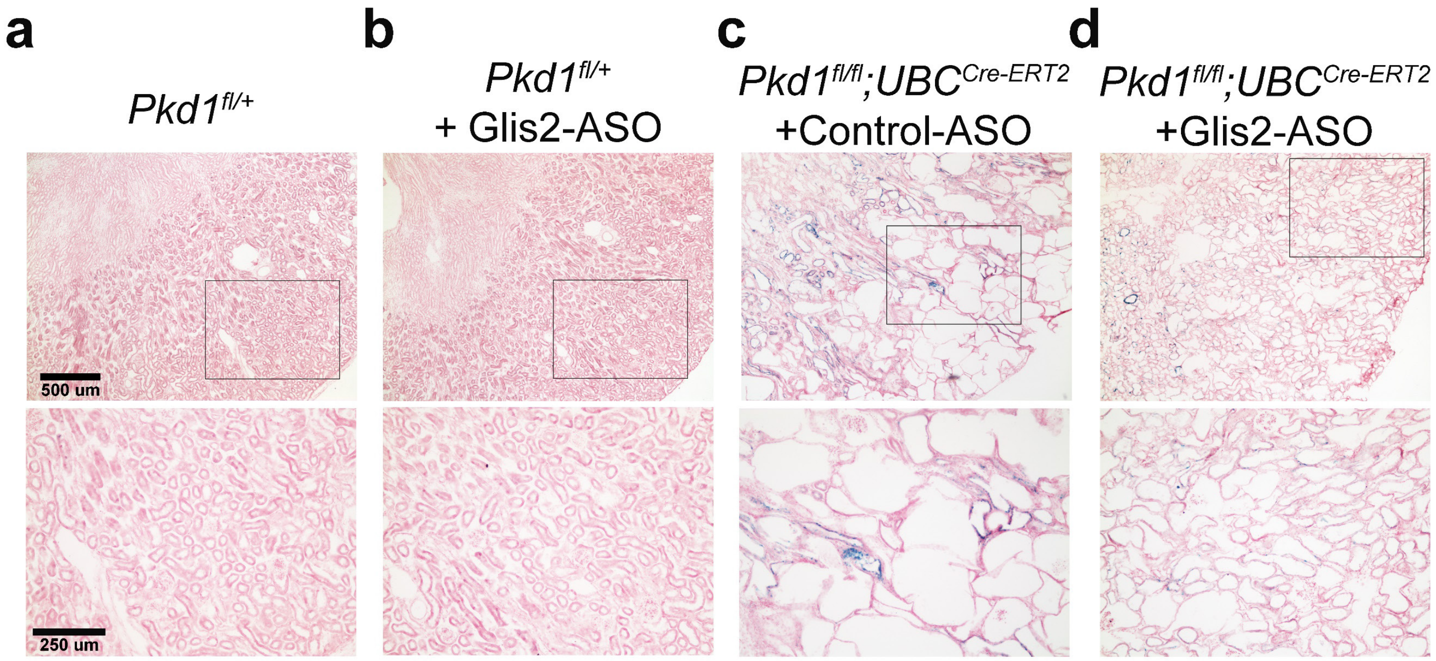

Supplementary Figure 24. *Glis2* ASO reduces cellular senescence in adult onset ADPKD. a-d, Representative bright-field microscopy images of kidney sections showing senescence-associated β galactosidase (SA-β-Gal) activity with the specified genotypes and treatments at 18 weeks age with lower (*upper panel*) and higher (*lower panel*) magnification. **a,b**, Blue staining indicative of cellular senescence is absent from noncystic kidneys, including those treated with Glis2-ASO (**b**). Evidence of cellular senescence is present sporadically in polycystic $Pkd1^{fl/fl};UBC^{Cre\text{-}ERT2}$ kidneys treated with control-ASO (**c**) and is qualitatively reduced following treatment with Glis2-ASO (**d**). At least one histological section from two mice for each genotype and treatment group were examined to identify representative images. Boxed regions in upper image are shown in the respective lower panels. Scale bars: 500 µm (*upper panels*); 250 µm (*lower panels*).
