# Supplementary material for: Glis2 is an early effector of polycystin signaling and a target for therapy in polycystic kidney disease
Source: Nat Commun. 2024 May 1;15:3698. doi: 10.1038/s41467-024-48025-6 (PMC11063051; doi:10.1038/s41467-024-48025-6)
Supplement: Supplementary file 3 — Description of Additional Supplementary Files [file 41467_2024_48025_MOESM3_ESM.docx]

**Description of Additional Supplementary Files**

**Supplementary Data Legends:**

**Supplementary Data 1: TRAP RNASeq quality metrics.**

**Supplementary Data 2: DEG analysis in male and female samples.** Gene annotation data, mean transcripts per million (TPM) values for each genotype and DEG for comparisons Pkd1^KO^ vs. noncystic, Pkd1^KO^ vs. Pkd1^KO^+cilia^KO^, and Pkd1^KO^+cilia^KO^ vs. noncystic in male mice. DEGs are ordered based on descending adjusted P value for Pkd1^KO^ vs. noncystic. Contents of sheets: *SKO.Centered.DEGs.male*, 15,991 genes; *FDR005&sameDirectionMale*, subset of DEG with the same relative direction of change in comparisons of Pkd1^KO^ vs. noncystic and Pkd1^KO^ vs. Pkd1^KO^+cilia^KO^ with FDR<0.05 (440 genes); *overlap_with_female*, subset of genes shared between male and female *FDR005&sameDirection* group (167 genes). *SKO.Centered.DEGs.female*, 15,968 genes; *FDR005&sameDirectionFemale*, subset of DEG with the same relative direction of change in comparisons of Pkd1^KO^ vs. noncystic and Pkd1^KO^ vs. Pkd1^KO^+cilia^KO^ with FDR<0.05 (526 genes); *overlap_with_male*, subset of genes shared between male and female *FDR005&sameDirection* group (167 genes). Detection of DEGs was done with the DESeq2 R package (version 1.30.1) using a negative binomial generalized linear model. The Benjamini-Hochberg procedure was used for multiple test correction with FDR <0.05 used as the threshold for statistical significance.

**Supplementary Data 3: Biologic pathway enrichment analysis using Metascape^1^**. Primary data corresponding to data presented in Supplementary Figure 4. Contents of sheets: *male*, statistically significantly enriched set of 440 genes with same direction in Pkd1^KO^ compared to both noncystic and Pkd1^KO^+cilia^KO^ in male mice corresponding to Supplementary Figure 4a; *female*, statistically significantly enriched set of 526 genes with same direction in Pkd1^KO^ compared to both noncystic and Pkd1^KO^+cilia^KO^ in female mice corresponding to Supplementary Figure 4b; *overlap*, statistically significantly enriched set of 167 genes with same direction in Pkd1^KO^ compared to both noncystic and Pkd1^KO^+cilia^KO^ in common between male and female mouse kidneys corresponding to Supplementary Figure 4c. The results of the enrichment analysis were obtained directly from Metascape^1^. Metascape uses the hypergeometric test to calculate P-values, which are then adjusted for multiple testing using the Benjamini-Hochberg correction algorithm.

**Supplementary Data 4: ‘CDCA pattern’ genes with broad expression along the nephron.** TRAP RNASeq DEG data cross-referenced with nephron segment-specific bulk RNASeq expression data^2^ to identify genes with TPM >1.0 in seven selected segments highlighted in yellow columns (proximal tubule S1, S2, medullary thick ascending limb, cortical thick ascending limb, distal convoluted tubule, connecting tubule, cortical collecting duct)^3^. Nephron segments definitions are presented in the “Legend” tab. The segment specific TPM presented are from published data in normal microdissected nephrons^3^. In addition, the mean TPM from TRAP RNASeq for each genotype and sex are presented in green highlighted columns. The last two columns show the number of microdissected segments for which the gene has TPM >1 in normal kidney (maximum 14) and the adjusted P value for the indicated comparison. Genes in the table were ordered first by the number of segments positive and then by adjusted P value. Selected genes discussed in the text are highlighted in red text. The P-values in this Table are obtained from Supplementary Data 2. The adjusted P-values in column U in the "MaleDEGs TPM >1" sheet are derived from the SKOvsWT comparison of 7-week male samples in Supplementary Data 2. The adjusted P-values in column U on the "FemaleDEGs TPM >1" sheet are obtained from the SKOvsWT comparison of 7-week female samples in Supplementary Data 2. In the "Male-FemaleOverlap TPM >1" sheet, the adjusted P-values in column X originate from the SKOvsWT comparison of 7-week male samples in Supplementary Table 2.

**Supplementary Data 5: Primer and probe sequences and antibodies. a,** Primer sequences for mouse genotyping; **b**, antibodies used in the studies; **c**, qRT-PCR primer sequences; **d**, single molecule fluorescence in situ hybridization (smFISH) probe sequences.

**References**

1. Zhou Y*, et al.* Metascape provides a biologist-oriented resource for the analysis of systems-level datasets. *Nature Communications* **10**, 1523 (2019).

2. Chen L, Chou CL, Knepper MA. A Comprehensive Map of mRNAs and Their Isoforms across All 14 Renal Tubule Segments of Mouse. *J Am Soc Nephrol* **32**, 897-912 (2021).

3. Ma M, Tian X, Igarashi P, Pazour GJ, Somlo S. Loss of cilia suppresses cyst growth in genetic models of autosomal dominant polycystic kidney disease. *Nat Genet* **45**, 1004-1012 (2013).

**Supplementary Movie Legends**

**Supplementary Movie 1.** **Glis2 is not detectable in primary cilia of IMCD3.** IMCD3 cells expressing Glis2-EGFP and Nphp3^(1-200)^-mApple were live imaged using confocal microscope to create a z-stack. Z-stack was used to create a movie with manual rotation to show Glis2 localization.

**Supplementary Movie 2.** **Glis2 is not detectable in primary cilia of IMCD3-Cas9**. IMCD3-Cas9 cells expressing Cas9, Glis2-EGFP and Nphp3^(1-200)^-mApple were live imaged using confocal microscope to create a z-stack. Z-stack was used to create a movie with manual rotation to show Glis2 localization.

**Supplementary Movie 3.** **Loss of *Pkd1* does not change Glis2 localization**. IMCD3-3F6 (*Pkd1*^-/-^ clone) cells expressing Cas9, Glis2-EGFP and Nphp3^(1-200)^-mApple were live imaged using confocal microscope to create a z-stack. Z-stack was used to create a movie with manual rotation to show Glis2 localization.
