# Supplementary material for: Glis2 is an early effector of polycystin signaling and a target for therapy in polycystic kidney disease
Source: Nat Commun. 2024 May 1;15:3698. doi: 10.1038/s41467-024-48025-6 (PMC11063051; doi:10.1038/s41467-024-48025-6)
Supplement: Supplementary file 8 — Supplementary Data 5 [file 41467_2024_48025_MOESM8_ESM.docx]

**a. Mouse allele genotyping primer sequences**

The primers for genotyping the mouse alleles are presented in the table below. We note that we present PCR genotyping primers for the *Pkd2^WS25^* allele which was previously only genotyped by Southern blotting^1^. The same forward primer is combined with the *Pkd2^WS25^* reverse primer to detect the *Pkd2^WS25^* allele with the *Pkd2^▬^* reverse primer which detects the *Pkd2* null allele in a manner that distinguishes it from *Pkd2^WS25^*.

Genotyping primers:

| **Gene** | **Forward Primer** | **Reverse Primer** |
| --- | --- | --- |
| *Pkhd1^Cre^* | 5’-CTGGTTGTCATTGGCCAGG | 5’-GCATCGACCGGTAATGCAGGC |
| *Pax8^rtTA^* | 5’-CCATGTCTAGACTGGACAAGA | 5’-CTCCAGGCCACATATGATTAG |
| *TetO^Cre^* | 5’-GCAGAGCTCGTTTAGTGAAC | 5’-TCGACCAGTTTAGTTACCC |
| *Pkd1^fl^* | 5’-CACAACCACTTCCTGCTTGGTG | 5’-CCAGCATTCTCGACCCACAAG |
| *Pkd2^fl^* | 5’-GGGTGCTGAAGAGATGGTTC | 5’-TCCACAAAAGCTGCAATGAA |
| *Tulp3^fl^* | 5’-CCATTTGTGAGGGTTGCTTT | 5’-GTTTTGTGCTGGGGATGGTA |
| *Ift88^fl^* | 5’-GACCACCTTTTTAGCCTCCTG | 5’-AGGGAAGGGACTTAGGAATGA |
| *Kif3a^fl^* | 5’-TCTGTGAGTTTGTGACCAGCC | 5’-GGTGGGAGCTGCAAGAGGG |
| *Glis2^fl^* | 5’-ACTGGGGGTAGGGTCCTAGA | 5’-CCCTGTCACCATCTCCAACT |
| *UBC^Cre-ERT2^* | 5’-GACGTCACCCGTTCTGTTG | 5’-AGGCAAATTTTGGTGTACGG |
| *ACTB^Cre^* | 5’-AGGTTCGTTCACTCATGGA | 5’-TCGACCAGTTTAGTTACCC |
| *ACTB^Flp^* | 5’-CACTGATATTGTAAGTAGTTTGC | 5’-CTAGTGCGAAGTAGTGATCAGG |
| *R26^Rpl10a^* (knockin) | 5’-CGTGTTCGTGCAAGTTGAGT | *5’-*ATTGCATCGCATTGTCTGAG |
| *R26^Rpl10a^* (WT) | 5’-CGTGTTCGTGCAAGTTGAGT | 5’-CCGAAAATCTGTGGGAAGTC |
| *Pkd2^WS25^* | 5’-GTGCTACTTCCATTTGTCACGTCCTGC | 5’-CTGATTCACATGCCCCAGGT |
| *Pkd2^▬^*  with *Pkd2^WS25^* | 5’-GTGCTACTTCCATTTGTCACGTCCTGC | 5’-GGGAAAACCATGGAAAATCG |
| *Glis2^+^* (WT) | 5’-CAGTCTGCCTGGCCTGCCATTAC | 5’-CAGACCCTGGTGGAATGACCAC |
| *Glis2^▬^* | 5’-TTCTATCGCCTTCTTGACGAG | 5’-GCGCCTTTCCAATGAGTCTTC |
| *Glis2^Ex3+^* (WT) | 5’-ACTGGGGGTAGGGTCCTAGA | 5’-CCCTGTCACCATCTCCAACT |
| *Glis2^Ex3▬^* | 5’-tcgtatagcatacattatacgaa | 5’-AATAGCGAAGTGGCTGGAAA |
| *ROSA^mT/mG^* (knockin) | 5’-TAGAGCTTGCGGAACCCTTC | 5’-CTTTAAGCCTGCCCAGAAGA |
| *ROSA^mT/mG^* (WT) | 5’-AGGGAGCTGCAGTGGAGTAG | 5’-CTTTAAGCCTGCCCAGAAGA |

**b. Antibodies**

| **Target** | **Antibody name** | **Company** | **Cat. no.** | **Dilution (IB)** | **Dilution (IF)** |
| --- | --- | --- | --- | --- | --- |
| PC1 | 7e12 | Santa Cruz Biotechnology | sc-130554 | 1:1000 | NA |
| PC2 | YCC2 | ^2^ | NA | 1:2000 | NA |
| GLIS2 | YNG2 | This study | NA | 1:40,000 | 1:1000 |
| KIF3a | D7G3 | Cell Signaling Technology | 8507S | 1:1000 | NA |
| IFT88 | IFT88 Polyclonal antibody | Proteintech | 13967-1-AP | 1:1000 | 1:500 |
| TULP3 | Tulp3 Polyclonal antibody | Proteintech | 13637-1-AP | 1:1000 | 1:500 |
| ZO1 | ZO1 Polyclonal antibody | Proteintech | 21773-1-AP | NA | 1:500 |
| ZO1 | ZO-1 Monoclonal antibody | Proteintech | 66452-1-Ig | NA | 1:500 |
| Acetylated α-Tubulin | AF647 | Santa Cruz Biotechnology | sc-23950 | NA | 1:1000 |
| ARL13b | ARL13b Mouse Monoclonal Antibody | Proteintech | 66739-1-Ig | NA | 1:1000 |
| HSP90 | HSP90 Antibody #4874 | Cell Signaling Technology | 4877S | 1:1000 | NA |
| LAMIN A/C | Lamin A/C Antibody #2032 | Cell Signaling Technology | 2032S | 1:1000 | NA |
| LAMIN B1 | Lamin B1 Polyclonal antibody | Proteintech | 12987-1-AP | 1:1000 | NA |
| Anti-FLAG | Mouse monoclonal ANTI-FLAG® M2 antibody | Sigma-Aldrich | F1804 | 1:500 | NA |
| Caspase-1 | Mouse anti-cleaved caspase-1 | Adipogen International | AG-20B-0042-C100 | 1:1000 | 1:200 |
| TNFα | Mouse anti-TNFα | Santa Cruz Biotechnology | SC-52746 | 1:1000 | 1:200 |
| α-SMA | Rabbit anti-α-SMA | Abcam | ab5694 | 1:1000 | 1:200 |
| PDGFR-β | Rabbit anti- PDGFR-β | Abcam | ab32570 | 1:1000 | 1:200 |
| F4/80 | Rat anti-F4/80 | AbD Serotec | MCA497R | NA | 1:200 |
| Ki-67 | Rabbit monoclonal anti-Ki-67 | Thermo Fisher Scientific | RM 9106-S1 | NA | 1:200 |
| Megalin | Rabbit anti-megalin | ^3^ | NA | NA | 1:500 |
| Dolichos biflorus agglutinin (DBA) | FITC DBA  or  Rhodamine DBA | Vector Laboratories | FITC (FL-1031)  Rhodamine (RL-1032) | NA | 1:50 |
| Lotus tetragonolobus agglutinin (LTA) | FITC LTA | Vector Laboratories | FL-1321 | NA | 1:200 |
| Secondary antibodies | Alexa-488, Alexa-594 and Alexa-647 conjugated secondary antibodies | Molecular ProbesThermo FIsher |  | NA | 1:500 |
| Hoechst 33342 | Nuclear stain | Molecular ProbesThermo Fisher | H-3570 | NA | 1:5000 |

**c. qRT-PCR primer sequences**

| **Gene** | **Forward Primer** | **Reverse Primer** |
| --- | --- | --- |
| *Pkd1* | 5’-CTAGACCTGTCCCACAACCTA | 5’-GCAAACACGCCTTCTTCTAATGT |
| *Pkd2* | 5’-GGGGAACAAGACTCATGGAAG | 5’-GCCGTAGGTCAAGATGCACAA |
| *Glis2* | 5’-GTGTCGATGGGCCAAGTGTAA | 5’-TGACAGCAGTATCGAGCATCC |
| *Kif3a* | 5’-ATGCCGATCAATAAGTCGGAGA | 5’-GTTCCCCTCATTTCATCCACG |
| *Cables2* | 5’-AGGAGACGAGTCACATCTCAG | 5’-TGTGTCATACTGCCTCATGTTC |
| *Chpf* | 5’-AGTGCCTGATGCCACCTATAC | 5’-CGGCCAAGATAGAGATGGGTTG |
| *Tspan5* | 5’-GGGAAGCACTACAAGGGTCC | 5’-CCACAGTCCGATTCCAAGAAA |
| *Anks3* | 5’-AGCTCAGCGATGAAGCCAG | 5’-CTGTGTGAAGATCCAAGGGGA |
| *Ptpdc1* | 5’-GGTCACTGACAATATCCTGGC | 5’-AGGTAGGTGAAACCACTTTCTTG |
| *Lad1* | 5’-ATGTCGGTCAGCAGAAAGGAC | 5’-CTGTGGTTGAACTCAGGTTGC |
| *Ntn4* | 5’-GCAGGCTTGAATGGAGTAGC | 5’-GCAGCGTTGCATTTATCACAC |
| *Gapdh* | 5’-AGGTCGGTGTGAACGGATTTG | 5’-TGTAGACCATGTAGTTGAGGTCA |
| *18s* | 5’-GTAACCCGTTGAACCCCATT | 5’-CCATCCAATCGGTAGTAGCG |
| *Adgre1* (F4/80) | 5’-AGTACGATGTGGGGCTTTTG | 5’-ACTCCTGGGCCTTGAAAGTT |
| *Cdkn1a* | 5’-AACATCTCAGGGCCGAAAAC | 5’-GTGGGCACTTCAGGGTTTTC |
| *Cdkn2a* | 5’-GGTCTTTGTGTACCGCTGG | 5’-CTGAGGCCGGATTTAGCTCT |

**d. Single molecule fluorescence in situ hybridization (smFISH) probe sequences**

| ***Glis2* Probe** | ***Glis2* Probe Sequence** |  | ***Lrp2* Probe** | ***Lrp2* Probe Sequence** |
| --- | --- | --- | --- | --- |
| Probe 1 | 5’-ttggtgatgctcagctttag |  | Probe 1 | 5’-cacagcgaaaattcccactg |
| Probe 2 | 5’-tcgatgcaaagcatgatgcc |  | Probe 2 | 5’-agtgtcatccaaacagtctc |
| Probe 3 | 5’-ggaaacctggtggtggagag |  | Probe 3 | 5’-tcggaacaatccttatcctg |
| Probe 4 | 5’-tcagggaatttggggttcag |  | Probe 4 | 5’-gtactcgatgggaacacact |
| Probe 5 | 5’-caagggggctgcagaaaagc |  | Probe 5 | 5’-aatagcagtttctctcgtca |
| Probe 6 | 5’-gagggtggtgacaagctgag |  | Probe 6 | 5’-gtcatggtcacagacatagg |
| Probe 7 | 5’-cagtaggcagtggaggcaag |  | Probe 7 | 5’-catcgctgttatcttcacag |
| Probe 8 | 5’-aacccaggggcaagaagaac |  | Probe 8 | 5’-ccattctcttgggtaacatg |
| Probe 9 | 5’-atcgacacaccagctgcttg |  | Probe 9 | 5’-cgggaactccatcacaaact |
| Probe 10 | 5’-gctcaaagagctggttacac |  | Probe 10 | 5’-ccaggatataaccttcttca |
| Probe 11 | 5’-tcgttgacatggtcaaccag |  | Probe 11 | 5’-tcgttggatttgcaatgctg |
| Probe 12 | 5’-ctgttcaggcttgacatgat |  | Probe 12 | 5’-caaatcccgaccattagaga |
| Probe 13 | 5’-ccaatgacagcagtatcgag |  | Probe 13 | 5’-tttcttccatgaagatctcc |
| Probe 14 | 5’-tcttgtacctggcattgaag |  | Probe 14 | 5’-atcaagggcaattcctctag |
| Probe 15 | 5’-tgtgtccggatgtgaatgag |  | Probe 15 | 5’-gattgctaccatccatgaag |
| Probe 16 | 5’-ttgtggatcttcaggttctc |  | Probe 16 | 5’-cgcttggatacaagatccag |
| Probe 17 | 5’-tggagtaacgcttgttgcag |  | Probe 17 | 5’-aaacggatgagggacgaggg |
| Probe 18 | 5’-tgtgcttgaagcggtcactg |  | Probe 18 | 5’-atggtaaactgtcactccga |
| Probe 19 | 5’-ttgtctacgtagtgggtacg |  | Probe 19 | 5’-cagccgtttgtgatgagaag |
| Probe 20 | 5’-cgggcatcttgcagtagtag |  | Probe 20 | 5’-agagattccttgagatccag |
| Probe 21 | 5’-tccgtgtaacgcttgtgaca |  | Probe 21 | 5’-cagcttcatgacagtgacac |
| Probe 22 | 5’-tgatgtgtttgcgcagtgaa |  | Probe 22 | 5’-tcgagggttgtttaagttgc |
| Probe 23 | 5’-atgtgacacaaagtggccat |  | Probe 23 | 5’-gactatgggcattaggtgag |
| Probe 24 | 5’-gacataggagccactgtcag |  | Probe 24 | 5’-aacggtgagtccaaacggat |
| Probe 25 | 5’-ttcgggatgatgatctgagc |  | Probe 25 | 5’-gtttggaactgggaagcaga |
| Probe 26 | 5’-gaggtagaggtaatggcagg |  | Probe 26 | 5’-ctctgaagcttcattccata |
| Probe 27 | 5’-cagagcactgaggtcaaggg |  | Probe 27 | 5’-ctgttgtcatggcaatcatc |
| Probe 28 | 5’-caaagggtgagggcaacagc |  | Probe 28 | 5’-cagtcattctgtttgtcaca |
| Probe 29 | 5’-aggagagaaaccacaggcag |  | Probe 29 | 5’-cactctttggggatacacat |
| Probe 30 | 5’-tgtggacaggtccagtacag |  | Probe 30 | 5’-ctgatccatccgaacaatca |
| Probe 31 | 5’-gagttaacacctctggactg |  | Probe 31 | 5’-cttgaactgagagcttgtgc |
| Probe 32 | 5’-acagaccctggtggaatgac |  | Probe 32 | 5’-tagatgcagtttccattgtc |
| Probe 33 | 5’-tttaccacagctggtttgag |  | Probe 33 | 5’-aaaaggctgggtaggacagt |
|  | | | Probe 34 | 5’-tttgtcacgactggctacaa |
|  |  |  | Probe 35 | 5’-tttaccctccaataagtcac |
|  |  |  | Probe 36 | 5’-ttgtcggttccgttttgaaa |
|  |  |  | Probe 37 | 5’-tatccagtctactgcaatca |
|  |  |  | Probe 38 | 5’-tctgtccagtaaatgttgcg |
|  |  |  | Probe 39 | 5’-tacattgtcacccattctag |
|  |  |  | Probe 40 | 5’-ccatgaagtagatgaggcga |
|  |  |  | Probe 41 | 5’-tggggaacgctgtacattac |
|  |  |  | Probe 42 | 5’-tcactctgtgaatttcacct |
|  |  |  | Probe 43 | 5’-ggagtgtcaacacctcaatg |
|  |  |  | Probe 44 | 5’-taatcagggttttcccatac |
|  |  |  | Probe 45 | 5’-taaaggaaggagccgtggac |
|  |  |  | Probe 46 | 5’-attggaagagtcagcagcat |
|  |  |  | Probe 47 | 5’-aggcagcatggaaacgacta |
|  |  |  | Probe 48 | 5’-gtgatcagacaactccaagc |

**References**

1. Wu G*, et al.* Somatic inactivation of Pkd2 results in polycystic kidney disease. *Cell* **93**, 177-188 (1998).

2. Cai Y*, et al.* Identification and characterization of polycystin-2, the PKD2 gene product. *JBiolChem* **274**, 28557-28565 (1999).

3. Zou Z, Chung B, Nguyen T, Mentone S, Thomson B, Biemesderfer D. Linking receptor-mediated endocytosis and cell signaling: evidence for regulated intramembrane proteolysis of megalin in proximal tubule. *J Biol Chem* **279**, 34302-34310 (2004).
